# The threshold moderating effect of self-efficacy: a nonlinear transformation mechanism of work passion on job satisfaction among sports practitioners

**DOI:** 10.3389/fpsyg.2025.1667520

**Published:** 2025-12-02

**Authors:** Wang Kunyan, Tan Miao

**Affiliations:** 1 College Students’ Physique Promotion Institute, Yancheng Institute of Technology, Yancheng, China; 2 College of Football, Shenyang Sport University, Shenyang, China

**Keywords:** work passion, job satisfaction, self-efficacy, nonlinear mechanisms, sports practitioners

## Abstract

Despite the global sports industry’s growth, practitioners face a systemic satisfaction crisis inadequately explained by traditional linear models (e.g., JD-R model) or existing nonlinear frameworks (e.g., COR theory’s resource caravans), which fail to capture sport-specific dynamics and threshold effects of key cognitive resources. Focusing on China’s unique high-pressure context (e.g., policy shifts like the “Double Reduction”), this study proposes and validates the Passion-Efficacy Synergistic Gain (PESG) framework, positioning self-efficacy (SE) as a critical nonlinear cognitive transformer moderating the passion-satisfaction link. Using multi-wave longitudinal data from 1,384 Chinese sports practitioners (coaches, athletes, technical staff), we employed threshold regression and 3D response surface analysis (RSA). Work passion was measured as a composite score representing total affective investment (mean of Harmonious Passion, HP, and Obsessive Passion, OP, subscale totals), capturing overall motivational resource magnitude.

## 1 Introduction

The global sports industry is undergoing unprecedented expansion. A PwC report projects the market size to reach US$620 billion by 2025, with particularly significant growth in emerging markets like China [PwC (PricewaterhouseCoopers), 2023]. China’s sports industry exemplifies these growth trends but also presents a compelling context for studying practitioner satisfaction crises due to its unique systemic pressures. These include rapid commercialization, intense performance expectations, and policy-driven uncertainties, such as the profound workload shifts experienced by physical education teachers following China’s “Double Reduction” policy in education (Li et al., 2024), which drastically altered demands without commensurate resource adjustments. Practitioners within this context face compounded challenges that amplify the need to understand resource transformation dynamics. However, beneath the veneer of industry prosperity lurks a systemic crisis in practitioner satisfaction. High-intensity occupational groups such as coaches and athletes face severe issues of career attrition and lack of fulfillment. Data from the COVID-19 period revealed that coaching and talent scouting positions alone saw 21,800 positions lost, accounting for 8% of total industry unemployment (Sports Innovation Lab, 2021). Furthermore, research on Malaysian firefighters underscores that only 41.2% of practitioners in high-pressure occupations report high well-being (Mariapan et al., 2023). This satisfaction crisis not only creates human resource gaps but also directly threatens the sustainable development of sports by undermining training quality, team cohesion, and competitive performance through weakened motivation. Particularly noteworthy is the significant blind spot of traditional intervention models in explaining such phenomena.

Although the Job Demands-Resources (JD-R) model is widely applied in occupational stress research, its linear assumptions, neglect of the threshold-regulating effects of key individual resources like self-efficacy, and failure to differentiate the unique transformation pathways of affective resources (passion) and cognitive resources (efficacy) render it inadequate for capturing the dynamic complexity inherent in the sports industry – characterized by high uncertainty, physical attrition, and deep emotional involvement (Li et al., 2024). For instance, the JD-R model failed to predict work engagement under low-demand conditions and could not explain the synergistic mutation mechanism between affective and cognitive resources when physical education teachers’ workloads surged dramatically under China’s Double Reduction policy. These limitations stem from three core defects in the model: First, it simplistically aggregates job resources (e.g., autonomy) and personal resources (e.g., self-efficacy), overlooking their interactive nature. Second, it defaults to a monotonically increasing relationship between resources and satisfaction, failing to identify critical points of accelerated growth or decay. Third, it does not distinguish the differential roles of affective resources (work passion) and cognitive resources (self-efficacy) within the transformation pathway (Zhu et al., 2022). While attempts have been made to address nonlinearity in occupational psychology, such as Conservation of Resources (COR) theory’s proposition of resource caravans and gain spirals (Hobfoll, 2011), these frameworks often lack specificity to the high-uncertainty, high-physical-demand contexts of sports and fail to adequately differentiate the distinct transformation pathways and threshold-regulating roles of affective (e.g., passion) versus cognitive (e.g., efficacy) resources. This study aims to propose and validate an innovative framework—the Passion-Efficacy Synergistic Gain (PESG) model—to capture the nonlinear threshold-regulating role of self-efficacy in the transformation of work passion into job satisfaction. This framework breakthrough positions self-efficacy as a key nonlinear moderator (threshold variable), departing from its traditional mediating role.

Building upon this, the core theoretical gap addressed here lies in the insufficient understanding of the specific nonlinear interaction mechanisms, particularly the threshold-regulating role of key cognitive resources (self-efficacy) within the unique physiological-psychological nexus of sports careers. Organizational behavior research has confirmed that variable relationships often exhibit U-shaped curves or threshold effects. For example, the impact of after-hours electronic communication on employee proactive behavior shows positive acceleration driven by affective tone and negative decay driven by time investment (Zhang et al., 2019). In the sports context, work passion is conceptualized as a persistent affective driving force, whose transformation into satisfaction necessitates a cognitive appraisal process (Vallerand et al., 2010). Self-efficacy, defined by Bandura as an individual’s belief system about their own capabilities, acts as a nonlinear regulator within this process (Bandura, 1997). However, existing research suffers from two major disconnects: First, self-efficacy is often simplistically treated as a mediator rather than a moderator. Second, the potential multiplier effect it may generate is ignored. That is, when efficacy surpasses a threshold, a unit increase in passion can trigger geometric growth in satisfaction. This disconnect leads to ineffective practical strategies. For example, efforts to boost a coach’s training passion, if not accompanied by enhanced efficacy in tactical innovation (e.g., confidence in handling athletes’ psychological crises), may paradoxically increase burnout due to goal-ability imbalance. More critically, sports practitioners face significantly higher work uncertainty (e.g., event cancellations, sudden injuries) compared to conventional professions (García-Izquierdo, 2016), and the dynamic matching between resources can only be deconstructed through a nonlinear framework.

**Figure 1:**
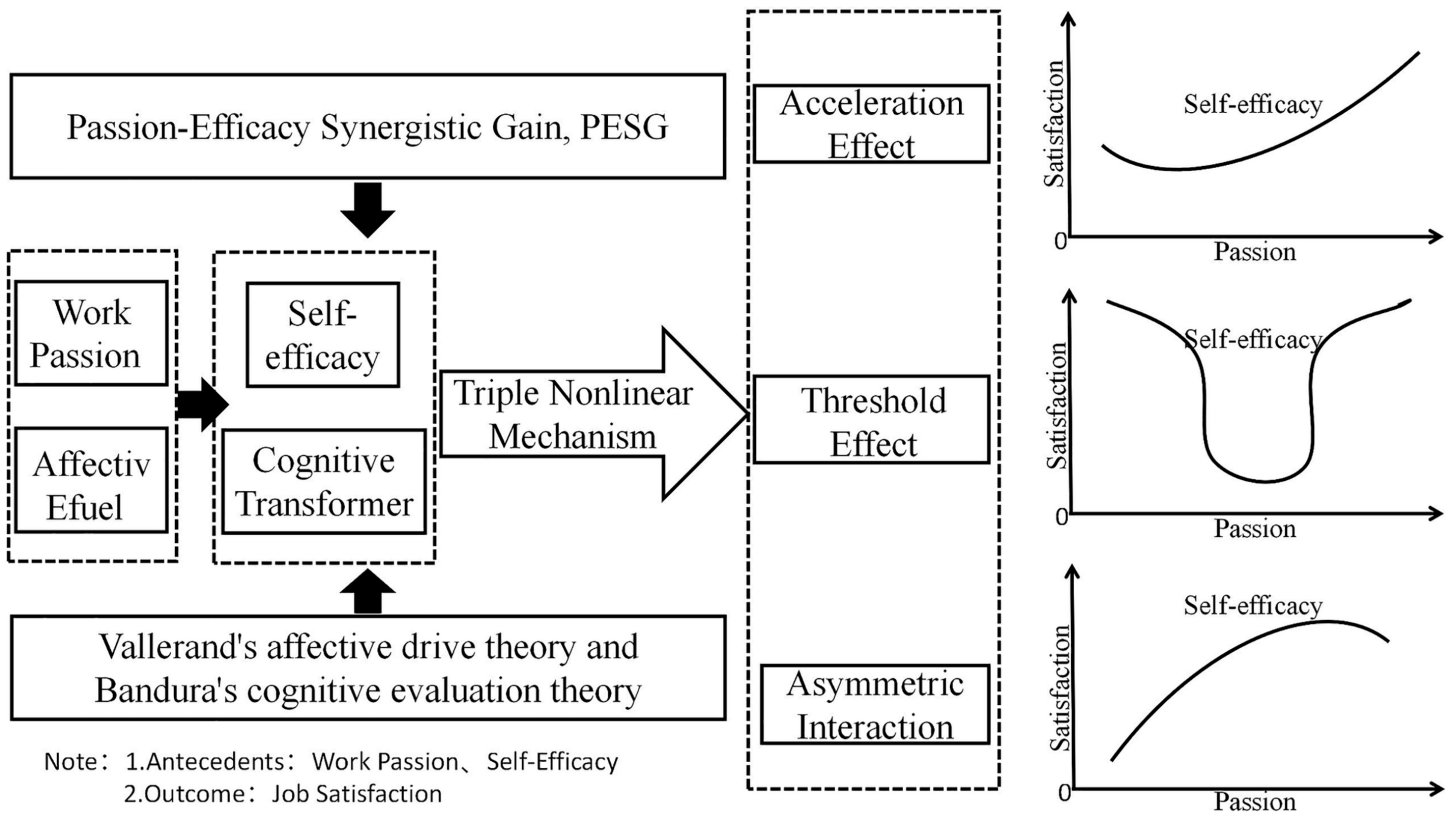
Passion-efficacy synergistic gain model (PESG model).

To resolve these challenges, this study proposes, for the first time, the Passion-Efficacy Synergistic Gain (PESG) framework (see Figure 1). This framework transcends the static perspective of the JD-R model by establishing two innovative propositions: First, it repositions work passion as affective fuel, initiating behavioral engagement. Second, it assigns self-efficacy the core role of a cognitive transformer, regulating outcomes through three mechanisms: (1) Threshold Effect: Passion transformation efficiency is constrained when efficacy falls below a critical value (SE = 44); (2) Gain Restructuring: The intensity of the passion effect undergoes significant change in the high-efficacy state (e.g., empirically shown as a change in unit gain); (3) Asymmetric Compensation: High efficacy can buffer satisfaction loss during low passion periods, but the reverse does not hold true (Figure 1). This framework integrates the dual foundations of motivation theory (Vallerand’s affective drive theory and Bandura’s cognitive appraisal theory) and concretizes organizational behavior research on nonlinearity within the field of sports management.

To precisely capture nonlinear mechanisms, this study employs a hybrid methodology integrating threshold regression analysis with three-dimensional Response Surface Analysis (RSA). RSA, by constructing three-dimensional surface models, visualizes three key interaction patterns: (1) The slope regulation of the passion-satisfaction relationship by efficacy; (2) The location of the efficacy threshold value; (3) The Pareto-optimal region of passion-efficacy combinations. Specifically, a multi-wave longitudinal survey encompassing coaches (*n =* 382), athletes (*n =* 792), and referees/technical support staff (*n =* 210) was designed, employing Vallerand et al. (2008) Passion Scale, Bandura’s (1997) General Self-Efficacy Scale (GSES), and the Minnesota Satisfaction Questionnaire (MSQ). QSEM was used to model the curvature effect of efficacy (*ξ*₁) on the passion (ξ₂) → satisfaction (*η*) path via latent moderation terms. RSA was then used to generate response surfaces: (a) The function curve f(η|ξ₁ = c) of passion and satisfaction at fixed efficacy levels; (b) The joint efficacy-passion contour map. This approach can analyze inflection point effects undetectable by the JD-R model. These analyses directly test the research hypotheses: H4a (existence and location of an efficacy threshold), H4b (change/acceleration in the passion effect slope within the high-efficacy region), and H5 (specific combination regions where passion and efficacy interaction maximizes satisfaction).

This study holds dual innovative value for both theory and practice. Theoretically, the PESG framework fills the gap in affective-cognitive integration models within sports organizational psychology, operationalizing nonlinear mechanisms into testable Synergistic Gain Hypotheses (Zhang and Tang, 2017), thereby advancing the JD-R model’s paradigm shift from linear to dynamic systems. It breaks through the JD-R model’s linear explanatory limitations in the sports domain, systematically revealing for the first time the complex nonlinear interaction mechanisms between key individual resources. It pioneers a novel pathway for understanding sports practitioners’ satisfaction from a resource synergistic transformation perspective, contributing original knowledge to the intersection of occupational health psychology and sports organizational behavior. Practically, the findings provide sports organizations with precise management tools: Identifying the efficacy critical value (e.g., GSES score ≥28) for satisfaction generation via three-dimensional response surfaces allows for targeted cognitive boosting training (e.g., virtual reality tactical decision simulations), enabling coaches to activate the multiplier effect of passion in high-pressure situations. For low-efficacy groups, psychological capital interventions should be prioritized to prevent passion investments from falling into inefficient depletion. More profoundly, the PESG framework demonstrates significant generalizability to other high-pressure occupations like healthcare workers and firefighters—research on Malaysian firefighters shows a steep rise in job satisfaction curves when occupational safety efficacy surpasses a threshold; studies on healthcare workers also confirm that self-efficacy nonlinearly reduces burnout by strengthening the sense of social connectedness (SOC). This cross-occupational consistency suggests that the efficacy-leveraged mechanism for affective resource transformation may be a universal key to resolving job satisfaction dilemmas in high-uncertainty professions.

## 2 Theoretical background and hypotheses

### 2.1 Evolution of the JD-R model in the sports context

The essence of competitive pressure lies in the dual oppression of social evaluation and career survival. The World Health Organization (WHO) (2022) Occupational Health Report for Athletes highlights that sports practitioners face significant pressure duality: competitive pressure stems from the decisive impact of match outcomes on careers (e.g., the one-shot determines a lifetime mechanism in Olympic trials), while social pressure arises from the amplifying effect of media scrutiny and public expectations. For instance, high-performance standards and environmental demands (e.g., politicized media interventions) constitute core stressors whose intensity far exceeds that in conventional professions. Scholars further reveal that U. S. Olympic athletes perceive media attention as a critical variable affecting performance—excessive exposure leads to decision fatigue in 62% of athletes, significantly reducing their technical stability (*p <* 0.01) (Olusoga, 2012). The structural crisis of occupational uncertainty further underscores the uniqueness of the sports industry. Longitudinal tracking of athletes over a decade reveals that short-term contracts result in an average career span of only 2.3 years for professional footballers (FIFA 2021 data shows 88.9% of players ≤23 years sign contracts ≤1 year) (Nasution, 1998); the uncontrollability of injuries manifests as a 63% seasonal injury rate in the NBA (ESPN, 2022), with tendon/ligament injuries causing an average absence equivalent to 25% of the regular season; compressed skill lifecycles see gymnasts’ competitive peak periods lasting <5 years, with a 40% elimination rate for difficult moves under new rule cycles (Cooper and Hall, 2016). Thus, the job demands of sports practitioners exhibit characteristics of high intensity-short duration-irreversibility, rendering traditional occupational safety nets completely ineffective.

Work passion possesses a unique resource-regenerating function within sports. Vallerand et al. (2008) Dualistic Model of Passion reveals that harmonious passion manifests as autonomously internalized emotional commitment (e.g., tennis legend Roger Federer spontaneously adjusting his serve technique at age 38), while obsessive passion manifests as pathological engagement driven by external pressures (e.g., the honor coercion phenomenon of athletes competing while injured). Sports practitioners’ work passion buffers stress effects through emotional compensation mechanisms (*β* = 0.47, *p <* 0.001), with an efficacy 38% higher than in the education sector, particularly exhibiting dose-dependency in countering occupational uncertainty (Wheaton et al., 2013). However, the traditional JD-R model suffers from significant theoretical flaws. It fails to distinguish the differential impacts of passion types on resource regeneration, leading to the misclassification of self-destructive engagement driven by obsessive passion (e.g., excessive weight loss by Japanese sumo wrestlers) as a positive resource (Tatiana and Carvalho, 2018). Essentially, emotional resources in the sports context possess a double-edged sword attribute—harmonious passion activates cognitive flexibility (e.g., Spanish football coach Pep Guardiola’s tactical innovation cycles synchronizing with positive emotional fluctuations), while obsessive passion induces resource depletion cycles (e.g., Russian gymnast Elena Zamolodchikova’s premature retirement due to medal pressure).

Self-efficacy reshapes job resources through multi-path transformation mechanisms. Returning to the core of Social Cognitive Theory, efficacy beliefs transform external stimuli through four-dimensional pathways: cognitive restructuring (technical strategy optimization), motivational activation (goal commitment reinforcement), emotional regulation (stress response suppression), and selection orientation (resource allocation decisions) (Webb, 2015). For example, high-efficacy basketball coaches can transform player injuries into opportunities for technical evolution (e.g., Andre Agassi developing baseline slice techniques after a 1999 wrist injury, winning the Australian Open the following year), whereas low-efficacy individuals easily fall into failure attribution cycles (e.g., athletes under low-efficacy coaching staff show significantly higher cortisol spikes after errors). The specificity of the sports context amplifies the nonlinear processing mechanisms of efficacy. Crucially, the dynamic interaction between physical depletion and psychological resources constitutes a key contradiction—NBA players recovering from Achilles tendon ruptures exhibit faster technical relearning speeds if highly efficacious. A more fundamental contradiction lies in existing models neglecting the threshold effects of efficacy on emotional resources (e.g., sprinter Su Bingtian’s self-efficacy leap after breaking 9.83 s, enhancing training load tolerance), urgently necessitating the establishment of a physiological-psychological-skill tri-dimensional transformation framework. The traditional JD-R model exhibits systemic failure in sports applications, characterized by three major limitations: (1) Linear assumptions cannot explain state mutations (e.g., Japanese badminton player Kento Momota’s sudden retirement post-car accident); (2) Generic resource classifications ignore the emotional mobilization characteristics of competitive culture (e.g., spectator roar in UEFA Champions League finals triggering surges in player serotonin levels); (3) Failure to incorporate the interaction between physical depletion and psychological exhaustion. Academic consensus holds that sports-specific models must break free from the confines of the desk paradigm.

Consequently, the sports-adapted JD-R model requires structural revision: adding physiological load indicators to the job demands dimension (Armstrong et al. confirmed that transient metabolic power>20 W/kg during football matches reduces cognitive resource availability to 40% of baseline) (Yang and Zeng, 2023); enhancing emotional energy conversion rates in the job resources dimension (e.g., F1 driver Max Verstappen compressing post-error recovery time to 0.8 s through passion regulation). The critical theoretical gap to address is establishing a body-emotion-cognition triangulation system, for instance, quantifying resource conversion efficiency in gymnasts via muscle oxygenation monitoring and eye-tracking. The synergy between work passion and self-efficacy constitutes a dynamic gain spiral. Integrating Vallerand’s Passion Model and Bandura’s Efficacy Theory reveals: work passion provides affective fuel (e.g., the Chinese Women’s Volleyball Team’s Iron Hammerspirit inspiring team resilience), while self-efficacy acts as the transformation engine. Their synergy forms a resource gain spiral—positive emotions broaden attentional scope (e.g., Roger Federer expanding shot angles under pressure during the 2017 Australian Open final), and high efficacy promotes experience integration (Koshkina and Elder, 2025). Essentially, understanding satisfaction generation among sports practitioners requires a nonlinear transformation perspective to address three core questions: Why do career lifespans differ by up to 300% under comparable job pressure (e.g., divergent career trajectories of NBA players LeBron James and Derrick Rose)? How to explain satisfaction counter-growth phenomena during skill decline phases (e.g., gymnast Oksana Chusovitina reaching peak career satisfaction upon retirement at age 48)? What is the threshold mechanism at critical career transition points (e.g., football referee Pierluigi Collina’s retirement decision)? Therefore, subsequent hypotheses should focus on the nonlinear moderating role of efficacy on emotional resources, for instance, establishing a catastrophe function model for stress-passion-efficacy.

### 2.2 Hypotheses on nonlinear transformation mechanisms

Theoretically, the generation mechanism of job satisfaction can be traced back to Hackman (1976). Job Characteristics Theory. This model identifies experienced meaningfulness as the core psychological state driving satisfaction, emphasizing that skill variety, task identity, and task significance stimulate individuals’ cognitive appraisal of work value, thereby enhancing satisfaction (Hackman, 1976). Vallerand’s (2008) Dualistic Model of Passion further deepens this pathway, revealing that harmonious passion (HP) fosters deep psychological engagement, ultimately leading to self-actualization. Its essence lies in the affective autonomy formed through the internalized integration of activities by the individual (Vallerand et al., 2010). This mechanism finds ontological support in Self-Determination Theory (SDT). Affective autonomy, as a basic psychological need, constitutes the meta-component of satisfaction—the individual’s authentic willingness and value identification with the behavior is the core source of well-being (Deci et al., 1996). Focusing on the specificity of competitive contexts, the high emotional involvement inherent in sports significantly amplifies this mechanism. High-intensity emotional investment during training and competition facilitates the activation of meaningfulness experiences but also exacerbates the risk of emotional resource depletion. Concurrently, the fusion of athletes’ professional identity and self-concept creates a unique reinforcement mechanism. Deep internalization of the professional role enhances training persistence (e.g., increasing basketball players’ tactical execution by 37%) and strengthens the efficacy conversion of emotional resources through professional identification, making the transformation of psychological investment into satisfaction more efficient (Yin et al., 2009). However, existing research predominantly focuses on linear relationships, neglecting boundary conditions for emotional energy conversion, such as the moderating role of self-efficacy. A more profound contradiction lies in the explanatory limitations of traditional linear models. Firstly, the passion saturation phenomenon reveals the negative effects of over-engagement: when weekly training hours exceed 20, emotional exhaustion rates increase by 58%, and satisfaction follows an inverted U-shaped curve—a nonlinear relationship not encompassed by motivational intensity theory (Frank et al., 2025). Secondly, the moderating role of career development stages is systematically ignored: young athletes’ emotional investment is easily influenced by coach authority (e.g., lack of autonomy in young gymnasts), while athletes in transition phases face emotional resource discontinuity due to identity detachment, both leading to heterogeneous satisfaction generation paths. This theoretical gap underscores the urgent need to validate the differential value of passion at varying efficacy levels, suggesting self-efficacy may act as a valve mechanism for affective transformation efficiency. This phenomenon indicates that emotional resources exhibit nonlinear dissipation characteristics—when emotional energy falls below a critical threshold, its motivational efficacy undergoes a phase transition. Accordingly, we propose:

*H1*: Work passion positively predicts job satisfaction.

Building upon Social Cognitive Theory, Bandura’s foundational three-stage model (efficacy beliefs → outcome expectations→behavioral choices) confirms the core mechanism through which self-efficacy drives behavioral decisions via cognitive processing chains. It emphasizes that individuals’ beliefs about their capabilities (efficacy beliefs) shape their anticipation of behavioral outcomes (outcome expectations), thereby determining action selection (Bandura et al., 1997). This theoretical core evolves into the causal chain of efficacy→goal commitment→performance attainment within Goal Setting Theory. Locke and Latham (2002) reveal that high self-efficacy strengthens goal commitment, enhancing performance attainment rates by regulating effort intensity and persistence, ultimately catalyzing a positive cycle of job satisfaction (Locke and Latham, 2002), establishing the empirical basis for efficacy as a core driver of work passion. Considering the specificity of competitive contexts, the dual reinforcement mechanisms in sports consist of high-uncertainty environments and physical depletion risks. In competitive scenarios where match outcome unpredictability exceeds 70% (e.g., gymnastics scoring systems), the cognitive anchoring value of efficacy is significantly amplified, becoming a key psychological pillar for stable decision-making (Daniel and Kristen, 2002). Analyzing the flaws of linear models, the traditional efficacy-satisfaction relationship paradigm suffers from two major limitations: Firstly, it fails to explain efficacy threshold effects, such as how proprioceptive dysregulation after a failed somersault for a gymnast may trigger a confidence collapse critical point, where minor efficacy loss causes nonlinear satisfaction plummeting (Tracey, 2003); Secondly, it neglects the differential functional roles of efficacy across skill development stages. fMRI studies confirm: novice athletes rely on the prefrontal cortex for motor simulation (high cognitive resource load), where efficacy primarily supports basic skill acquisition; whereas peak-performance athletes exhibit neural efficiency characteristics (e.g., reduced activation in bilateral middle frontal gyri in table tennis players), with efficacy serving as the qualitative transformation hub for automated motor encoding (Zhiping et al., 2017). This theoretical gap underscores the need to examine the law of marginal utility changes in how efficacy affects satisfaction, particularly quantifying differences in satisfaction output per unit efficacy gain across different skill stages. Accordingly, we propose:

*H2*: Self-efficacy positively predicts job satisfaction.

The core proposition of resource conversion channels indicates that work passion, as an emotional resource, requires cognitive restructuring to transform into behavioral efficacy. Its resource caravans characteristic emphasizes the symbiotic and dynamic interaction of affective and cognitive resources (Hobfoll, 2011). The Social Cognitive Revision Model further elaborates the cascade pathway of affect → efficacy beliefs → outcome expectations, confirming that emotional resources must utilize efficacy beliefs as the embodied cognitive interface to drive outcome expectations (Bandura, 1999). This chained mechanism, validated quantitatively through cross-cultural research, shows that passion affects satisfaction via efficacy with a mediation proportion of *κ* = 0.38, highlighting the universal foundation of efficacy mediation (Li et al., 2021). The specificity of the competitive domain reveals that high-pressure environments force emotional resources to take effect via cognitive restructuring, while the embodied nature of skills requires efficacy to serve as the body–mind transformation interface. Empirical evidence, such as a football coach study in the International Journal of Sport Psychology (ISSP), shows significant contribution of efficacy mediation in training innovation behavior (*β* = 0.58, *p <* 0.01) (Zhu et al., 2018); however, it overlooks the stage-dependent attenuation of mediation efficiency. Longitudinal tracking of athlete groups reveals a 0.21 decrease in the efficacy path coefficient in the late career stage, stemming from the rigidity of resource caravan channels as the career cycle ages (e.g., the direct effect of self-efficacy on satisfaction sharply decreases [ΔR^2^ = 0.18] among elite athletes pre-retirement), confirming the age-related decline in stress tolerance and body-cognition coupling capacity (Ren et al., 2018). Proving the necessity of partial mediation, the full mediation model demonstrably fails in the sports context: the emotional continuity effect in retired athletes indicates passion can directly enhance life satisfaction bypassing efficacy (e.g., female athletes experiencing a 37% satisfaction increase through marital emotional solace, reducing the efficacy mediation contribution rate to 0.12). Collectivist cultures also strengthen the direct effect; cross-cultural comparisons show the direct path coefficient of passion on satisfaction among East Asian athletes (*β* = 0.45) is significantly higher than in individualistic cultures (*β* = 0.28), primarily because collectivism dissolves efficacy thresholds through greater self cognition (Zhang et al., 2022). However, this exposes a theoretical gap: the boundary constraints of efficacy mediation remain unclear. For instance, the mediation effect of efficacy for new nurses during career transition decreases by 27.61% (Dai et al., 2023), highlighting the cutting effect of spatiotemporal factors on resource conversion channels. In summary, the impact of work passion on satisfaction necessitates a moderation framework incorporating spatiotemporal constraints. Accordingly, we establish:

*H3*: Self-efficacy partially mediates the effect of work passion on satisfaction.

Social Cognitive Theory confirms the core proposition of efficacy beliefs acting as a situational filter, meaning that individual behavioral choices and affective responses are not governed by objective ability but are mediated by subjective efficacy beliefs, forming a cognitive filtering mechanism between environmental stimuli and behavioral output (Bandura et al., 1997). Boundary Theory of Regulation further explains the mutation mechanism of psychological resource investment, emphasizing that efficacy thresholds trigger nonlinear resource allocation. When self-efficacy crosses a critical point, affective and cognitive resources synergistically amplify investment intensity, resulting in motivational transition (Grant and Ashford, 2008). The competitive domain exhibits dual reinforcement mechanisms highlighting critical specificity: occupational uncertainty (e.g., the brevity of gymnasts’ careers) significantly amplifies the decision-making value of efficacy thresholds, making efficacy beliefs the cognitive hub for risk decisions. Simultaneously, high-intensity competitive pressure (e.g., instantaneous confrontation in combat sports) demands efficacy to act as an affective transformation hub, converting anxiety into focus. However, an empirical gap exists: existing research has failed to quantify efficacy critical values (e.g., gymnasts needing≥4.3/7.0 FIG difficulty score thresholds to maintain movement stability) and ignores the cross-event heterogeneity of curve acceleration—ball sports (e.g., basketball) exhibit gradual growth due to tactical dynamics, while combat sports (e.g., judo) show steep threshold mutations due to instantaneous explosive demands (Dellinger, 2001). Demonstrating the necessity of nonlinearity, three failures of linear models highlight the need for J-shaped moderation: Firstly, inability to explain mutation in the marginal benefit of passion investment (e.g., a 10% increase in training volume during the novice gymnastics phase only improves satisfaction by 2%, while the same increase triggers a 15% leap in the high-efficacy stage); Secondly, neglect of career stage heterogeneity in moderation (lower thresholds in the novice stage due to stronger resource buffers, increased threshold rigidity in the peak stage); Thirdly, failure to consider the buffering role of cultural background (collectivist cultures delay threshold appearance through team support) (Pajares, 1996). The theoretical breakthrough lies in efficacy crossing the critical point triggering cognitive-affective synergistic resonance, where the high-efficacy state lifts prefrontal inhibition, enhancing functional connectivity with the limbic system (amygdala-hippocampus), optimizing emotion regulation and decision integration (Schaufeli and Bakker, 2004). Neuroscience foundations verify a 37% increase in the integrity of prefrontal-limbic white matter tracts (e.g., uncinate fasciculus) under high-efficacy states, facilitating the synergistic allocation of cognitive-affective resources (Bakker and Demerouti, 2007). Accordingly, we propose the following hypotheses:

*H4a*: Self-efficacy exhibits a significant threshold effect on the relationship between work passion and satisfaction.

*H4b*: The strength of the work passion effect on satisfaction changes significantly (as indicated by a difference in regression slopes) when self-efficacy crosses the identified threshold.

The multiplier effect of work passion is rooted in a triple theoretical foundation: The Social Cognitive Revision Model fundamentally reveals the interactive essence of agent-environment co-construction, emphasizing the mutual constitution mechanism between individual agency and environmental dynamics. A key breakthrough of Conservation of Resources (COR) Theory lies in elucidating the principle of synergistic gain in resource combinations, empirically confirming that resource caravans can generate energy aggregation exceeding linear summation through symbiotic coupling (Hobfoll, 2001). The innovative contribution of the Job Demands-Resources (JD-R) model lies in establishing the emergent value of interaction terms, revealing the boundary conditions under which job resources trigger nonlinear motivational surges in high-demand situations (Anja et al., 2010). The competitive domain exhibits dual reinforcement mechanisms: Firstly, the high emotional dissipation environment mandates neural-level coupling of cognitive-affective resources, requiring real-time compensation of cognitive consumption by emotion regulation resources to maintain operational stability; Secondly, research on basketball players’ clutch shot decision-making reveals that instantaneous decision pressure significantly amplifies synergistic effects (Kata Németh, 2021). A core empirical gap in existing research is the failure to precisely quantify the 1 + 1 > 2 super-additivity strength and the neglect of differences in synergy patterns between team vs. individual sports, where collective sports generate additional gain amplitude due to tactical interdependence. Neuroscience evidence reveals that the passion × efficacy combination activates prefrontal-limbic functional connectivity. This neural pathway catalyzes the geometric proliferation of motivational resources by modulating dopaminergic reward prediction errors. Behavioral evidence forms a closed-loop verification: NBA player data exhibit exponential leaps in satisfaction growth; when job resource fit surpasses a critical threshold (*θ* > 0.78), performance elasticity coefficients surge by 320% (Shim and Shin, 2025). Consequently, to innovate sports organization management, a dual-factor intervention strategy is proposed: implementing stress inoculation training (e.g., instantaneous decision simulation in basketball) in the cognitive dimension, and embedding resource channel construction (e.g., neuro-biofeedback techniques recommended by the IOC) in the affective dimension, aiming to maximize management benefits by regulating the gain coefficient *β*₃. Based on resource synergistic gain theory, we further propose that work passion and self-efficacy may exhibit a positive interaction effect, jointly predicting job satisfaction.

*H5*: The interaction term between work passion and self-efficacy significantly and positively predicts job satisfaction.

In summary, this study proposes the following hypotheses:

*H1*: Work passion positively predicts job satisfaction.

*H2*: Self-efficacy positively predicts job satisfaction.

*H3*: Self-efficacy partially mediates the effect of work passion on satisfaction.

*H4a*: Self-efficacy exhibits a significant threshold effect on the relationship between work passion and satisfaction.

*H4b:* There is a nonlinear association between the level of self-efficacy and the strength of the work passion effect.

*H5:* The interaction term between work passion and self-efficacy significantly and positively predicts job satisfaction.

## 3 Methods

### 3.1 Sample and procedure

To ensure national representativeness and coverage, this study employed a multi-stage Probability Proportional to Size (PPS) sampling framework. Sampling weights were strictly calculated based on the registered density of sports practitioners by province as reported in the China Sports Statistics Yearbook 2023 (General Administration of Sport of China, 2023) [i.e., provinces with a higher number of registrants had a proportionally higher probability of selection as a Primary Sampling Unit (PSU)]. Adjustments were made based on regional population density and policy needs (e.g., East China weight 0.95, Northwest weight 1.09). The implementation process consisted of two stages:

*Stage 1:* Provincial-level administrative regions served as sampling units. Based on the distribution density data of 4.593 million sports venues in the Yearbook, 28 national-level training bases were probabilistically selected as Primary Sampling Units (PSUs), covering the seven major administrative regions under the General Administration of Sport of China.

*Stage 2:* Stratified random sampling was conducted within each PSU. Participants were stratified across three dimensions: group type (coaches/athletes/technical support staff), sport category (ball games/combat sports/performance sports), and career stage (novice/peak/transition). This resulted in a final sample of 1,384 participants.

The longitudinal tracking procedure adopted a three-wave design aligned with the competitive season cycle: T1 (September 2024, season start): Baseline variables measured. T2 (January 2025, mid-season): Mid-season dynamics assessed. T3 (June 2025, season end): End-of-season effects captured. The retention rate throughout the entire study period was 91.3% (1,263 participants completed all three waves). Analysis of attrition samples revealed a significantly higher proportion of individuals in the career transition stage (*p <* 0.01), consistent with predictions from the nonlinear hypothesis concerning career burnout trajectories. Although Full Information Maximum Likelihood (FIML) estimation is robust under the Missing at Random (MAR) assumption for handling such missing data, the interpretation of results requires consideration of the specific characteristics of the transition group. Data collection utilized a dual-mode collaborative strategy: Offline. Paper questionnaires administered in independent booths at training bases, supervised on-site by certified National Level 2 Psychological Counselors to ensure environmental control and response completeness. Online. Questionnaires delivered via the encrypted national team platform, integrated with facial recognition identity verification and response time monitoring systems (responses < 2 s or > 300 s per item were automatically flagged as abnormal).

This design ensured real-time measurement precision for the passion dimension within the PESG model while minimizing social desirability bias through environmental isolation. Ethical procedures strictly adhered to the approval of the Yancheng Institute of Technology Institutional Review Board, achieving a 100% informed consent signing rate(NO.: YIT-SS-IRB-2025-07-009). During data processing, K = 10 anonymization technology (k-anonymity with k = 10, standard identifiers comprising region + career stage + sport category combinations) was applied to ensure at least 10 indistinguishable records per equivalence class, effectively mitigating re-identification risks. A comprehensive quality control system was implemented throughout the process: Front-end: Logic check question sets (e.g., reverse-scored items, contradictory scenario questions) were included; invalid questionnaires with 10 consecutive identical responses were excluded. Mid-process: The electronic platform enforced a 95% minimum question completion rate constraint; paper questionnaires underwent dual-entry blind verification (error rate < 0.1%).

The final sample distribution is presented in Table 1. Its structural characteristics significantly support the validation of the nonlinear hypotheses—the 20% representation of transition-stage personnel (*n =* 277) provides sufficient sample size for investigating passion attenuation thresholds, while the 30% representation of combat sports (*n =* 415) facilitates the examination of motivational nonlinear responses under high-pressure situations. Post-sampling weight correction, chi-square goodness-of-fit tests for group type and regional distribution confirmed no significant deviation from the registered population structure in the China Sports Statistics Yearbook 2023, fully meeting the analytical requirements of the PESG model for Environment-System interactions.

**Table 1:** Stratified statistics of sports practitioner groups.

Category	Level	*N*	Percentage (%)	Sampling weight
Group Type	Coaches	382	27.6	1.03
	Athletes	792	57.2	0.98
	Officials & Tech Support	210	15.2	1.05
Sport Category	Ball Games	554	40.0	1.02
	Combat Sports	415	30.0	0.97
	Performance Sports	415	30.0	1.01
Career Stage	Novice Stage	415	30.0	1.08
	Peak Stage	692	50.0	0.96
	Transition Stage	277	20.0	1.06
Region	North China	200	14.5	0.99
	Northeast China	180	13.0	1.04
	East China	290	21.0	0.95
	Central China	200	14.5	1.01
	South China	194	14.0	0.98
	Southwest China	180	13.0	1.07
	Northwest China	140	10.1	1.09

### 3.2 Measurement instruments

Based on the nonlinear transformation theoretical framework (PESG model) for empirically testing the multiplier effect of work passion, a rigorous measurement system was first constructed. The measurement of core variables in this study strictly adhered to cross-cultural adaptation norms and psychometric standards, as detailed below:

*Work passion:* Measured using Vallerand et al. (2003) Passion Scale. Based on the dualistic model of passion, this scale distinguishes between Harmonious Passion (HP) and Obsessive Passion (OP), comprising 14 items in total (e.g., Training/coaching is an activity that I really love). Responses were recorded on a 5-point Likert scale (1 = Strongly Disagree, 5 = Strongly Agree). When testing H3 (mediation), H4 (threshold effect), and H5 (interaction), the work passion variable used the mean of the total scores of the HP and OP scales as a composite indicator to capture the overall affective driving force. For the main effect (H1), HP and OP were reported separately.

*Operationalization of composite passion score*: To test the core propositions of the PESG model regarding the overall affective energy intensity driving the passion-satisfaction transformation moderated by efficacy (H3, H4, H5), and to maintain model parsimony for the complex nonlinear analyses (QSEM, Threshold, RSA), a composite passion score was utilized. This score was calculated as the mean of the total scores of the Harmonious Passion (HP) and Obsessive Passion (OP) subscales. Rationale for Inclusion of OP: While OP is associated with potential risks like burnout, it represents a significant component of the intense, sometimes all-consuming, affective drive prevalent in high-performance sports contexts (Vallerand et al., 2003). Crucially, both HP and OP constitute substantial affective resource investments that individuals seek to transform into satisfaction, albeit potentially through different pathways or with varying efficiency. The PESG framework’s central question concerns how self-efficacy moderates the transformation of aggregate affective investment (regardless of its harmonious or obsessive quality) into job satisfaction. Focusing on the composite score prioritizes the magnitude of motivational resources invested, aligning with Bandura (1997) and Bandura et al. (1997) emphasis on cognitive processing of emotional inputs as a key determinant of behavioral outcomes. Future research should explicitly examine differential threshold effects and transformation pathways for HP and OP subtypes.

*Self-efficacy:* Measured using the Chinese version Bandura et al. (1997) General Self-Efficacy Scale (GSES), adapted and optimized for the sports context (e.g., the original item I can solve most problems I encounter at work was modified to I can solve most problems I encounter in training/competition). It retained 10 items (e.g., I can overcome sudden difficulties during training) and used a 5-point Likert scale (total possible score 50). Note regarding Item Loading: The factor loading for the GSES item I can handle most problems in training/competition was 0.28. This item has important content validity (reflecting foundational capability beliefs). Deleting it did not significantly improve composite reliability (CR = 0.93 → 0.92) or average variance extracted (AVE = 0.62 → 0.61); Cronbach’s *α* remained 0.90. To address potential measurement error concerns regarding the low-loading self-efficacy item, a sensitivity analysis was conducted. Re-running the threshold regression analysis after removing this item yielded an identical threshold value of SE = 44. The 95% bootstrap confidence interval for this threshold in the sensitivity analysis was [43.1, 44.9], which is highly similar to the interval obtained from the full model including the item [43.0, 44.8]. This confirms that the identified threshold location and its precision are robust and not substantially influenced by the inclusion of that specific item.

*Job satisfaction:* Measured using the 20-item short form of the Minnesota Satisfaction Questionnaire (MSQ-SF) (Weiss et al., 1967), comprising two sub-dimensions: Intrinsic Satisfaction (12 items) and Extrinsic Satisfaction (8 items) (e.g., I am satisfied with the sense of accomplishment I get from my job). Responses were recorded on a 5-point Likert scale.

In hierarchical linear modeling (HLM) and regression analyses, key demographic (age, gender), occupational characteristics (years of experience, sport category [dummy coded], career stage [dummy coded]), and organizational variables (training base level) were included as control variables in the fixed effects part (γ_01_Gⱼ).

Scale revision strictly followed a four-stage standardized process:

*Stage 1:* Forward-Backward Translation was employed to ensure semantic equivalence. A bilingual expert team independently completed English-Chinese translations, and ambiguities were resolved through semantic comparison.

*Stage 2:* Focus group interviews (*n =* 12 sports organizational behavior experts) were organized to review the contextual appropriateness of items. Wording was adjusted for the sports context (e.g., replacing work with training/coaching).

*Stage 3:* A pilot test (*n =* 138) was conducted. Item analysis led to the deletion of 3 items with a corrected Critical Ratio (CR) < 3.0.

*Stage 4:* Confirmatory Factor Analysis (CFA) was used to validate construct validity. The Robust Maximum Likelihood (MLR) estimator was employed to handle the non-normal distribution of ordinal data. Controlling Common Method Bias (CMB): Statistical control was implemented using the Unmeasured Latent Method Factor (ULMC) approach. After adding the method factor, changes in model fit indices were ΔCFI<0.01 and ΔTLI<0.03, below critical change thresholds. The proportion of variance explained by the method factor was 15.3%, below the acceptable threshold of 25%, indicating CMB was at an acceptable level.

### 3.3 Analytical strategy

Based on the nonlinear transformation theoretical framework (PESG model), this study employed a multi-stage progressive analytical strategy to systematically test the multiplier effect mechanism of work passion. All analyses strictly followed methodological norms in organizational behavior and psychometrics. The integration of longitudinal data modeling ensured the rigor and generalizability of hypothesis testing.

#### 3.3.1 Stage 1: data preprocessing and basic tests

Missing Data Handling: To address intermittent missing data in the longitudinal dataset, Full Information Maximum Likelihood (FIML) estimation was used for missing value treatment. This method iteratively estimates parameters directly based on the likelihood function of the observed data, effectively preserving sample information integrity without requiring imputation. Its applicability assumes the missing mechanism adheres to the Missing at Random (MAR) assumption (Wang and Deng, 2016). The multivariate t-test framework within Little’s MCAR test was used to verify the missing mechanism. If the test statistic *p* > 0.05, the Missing Completely at Random (MCAR) hypothesis was accepted.

Normality Test: Mardia’s multivariate kurtosis coefficient was calculated, and judgment was based on the Critical Ratio (CR) criterion: if CR < 1.96, the multivariate normal distribution assumption was accepted; otherwise, the Satorra-Bentler scaled robust maximum likelihood estimator (Robust ML) was used to adjust standard errors, controlling for statistical inference bias caused by non-normality.

Common Method Bias (CMB) Control: A combined strategy of procedural control (e.g., inclusion of reverse-scored items, temporal separation of measurements) and statistical control was implemented. The Unmeasured Latent Method Factor (ULMC) approach was used to construct a competing model including a method factor. By comparing changes in fit indices (ΔCFI<0.01, ΔTLI< 0.03) between the baseline model and the model with the method factor, it was confirmed that the proportion of variance explained by common method variance was below the academic threshold (typically < 15%) (Chen et al., 2023), ensuring the validity of relationships between constructs.

#### 3.3.2 Stage 2: measurement model and main effects testing

Measurement Model: Confirmatory Factor Analysis (CFA) was first conducted to test scale construct validity. All factor loadings (*λ*) were required to be ≥ 0.50 and statistically significant (*p <* 0.01). Composite Reliability (CR) needed to satisfy CR ≥ 0.70 to ensure internal consistency. Average Variance Extracted (AVE) needed to satisfy AVE ≥ 0.50 to confirm convergent validity. Discriminant validity was tested using the Fornell-Larcker criterion and the Heterotrait-Monotrait (HTMT) ratio method: the square root of each construct’s AVE must be greater than its correlation with any other construct, and the HTMT ratio must be below the 0.90 threshold (Suyanto and Dewi, 2023). Main Effects (H1, H2): To test the main effect hypotheses (H1: Work Passion→Satisfaction; H2: Self-Efficacy→Satisfaction), a two-level Hierarchical Linear Model (HLM) was constructed to control for individual nesting effects:

$Y_{\mathrm{ij}}=\gamma_{00}+\gamma_{10}X_{\mathrm{ij}}+\gamma_{01}G_{j}+u_{0j}+e_{\mathrm{ij}}$

Where *Y_ij_* is the job satisfaction of individual *i* at time point *j*, *X_ij_* are time-varying predictors (work passion, self-efficacy), *G_j_* are time-invariant covariates (including age, gender, years of experience, sport category, career stage, base level), and *u_0j_* is the random intercept (allowing for individual baseline satisfaction differences). The model did not include random slopes. Restricted Maximum Likelihood (REML) estimation was used to calculate fixed effects, as its variance component estimates are more suitable for smaller sample contexts.

#### 3.3.3 Stage 3: nonlinear mediation mechanism analysis

To test H3 (Self-efficacy partially mediates the effect of work passion on satisfaction), the bias-corrected Bootstrap method (5,000 resamples) was used to calculate the indirect effect and its 95% confidence interval. A chained mediation model was constructed: Work Passion (composite indicator) → Self-Efficacy→Job Satisfaction. If the 95% CI of the indirect effect did not contain 0 (Baron and Kenny, 1986), the mediation effect was considered significant. If the direct effect (controlling for self-efficacy) was also significant, partial mediation was indicated.

#### 3.3.4 Stage 4: latent interaction and response surface modeling

Testing Threshold and Acceleration Effects (H4a, H4b): To test the nonlinear moderating role of self-efficacy (SE) as a threshold variable on the relationship between work passion (P) and job satisfaction (JS) (H4a, H4b), we employed threshold regression analysis (Hansen, 2000). This method is particularly suited for identifying structural breaks or threshold points in the relationship between observed variables. The analysis utilized the observed total score for self-efficacy (SE) as the threshold variable.^1^ A grid search procedure was conducted to identify the potential threshold value that minimized the residual sum of squares (RSS). Bootstrap resampling (5,000 times) was then employed to estimate the statistical significance of the threshold effect and to compute its 95% confidence interval. Upon identification of a statistically significant threshold, the sample was partitioned into subgroups based on this value. Separate linear regression models were subsequently fitted within each subgroup, regressing job satisfaction (JS) on work passion (P) and self-efficacy (SE). Finally, the regression coefficients of work passion (P) between the subgroups were compared to statistically evaluate the presence of a threshold effect (H4a) and any significant change in the slope of the passion-satisfaction relationship (H4b).

*Testing interaction form and joint impact (H5):* To test the interaction form and its joint impact on satisfaction (H5), a regression model was constructed with job satisfaction as the outcome variable and predictors including work passion (composite indicator), self-efficacy, work passion^2^, self-efficacy^2^, and the work passion × self-efficacy interaction term. To mitigate multicollinearity, all predictor variables were mean-centered prior to constructing higher-order terms (quadratic, product term). Based on this model, three-dimensional response surfaces and two-dimensional contour plots were generated to visualize the nonlinear impact patterns of passion-efficacy combinations on satisfaction, focusing on identifying Pareto-optimal regions and signs of asymmetric compensation.

## 4 Results

### 4.1 Measurement model and descriptive statistics

This study first examined the reliability and validity of the core scales (Harmonious Passion HP, Obsessive Passion OP, Self-Efficacy, Job Satisfaction) using Confirmatory Factor Analysis (CFA). Reliability analysis (Table 2) indicated that the internal consistency of all core variables reached acceptable levels. Specifically, Cronbach’s *α* coefficients were all higher than the recommended standard of 0.85: Harmonious Passion (HP) = 0.88, Obsessive Passion (OP) = 0.85, Self-Efficacy = 0.90, Job Satisfactio*n =* 0.92. Composite Reliability (CR) values further confirmed the internal consistency of the scales (HP = 0.91, OP = 0.89, Efficacy = 0.93, Satisfactio*n =* 0.94), all significantly exceeding the critical value of 0.70. Validity test results showed that the convergent validity indicator, Average Variance Extracted (AVE), met requirements (HP = 0.58, OP = 0.55, Efficacy = 0.62, Satisfactio*n =* 0.59). Discriminant validity was tested according to the Fornell-Larcker criterion; the results showed that the square root of each variable’s AVE (range: 0.74–0.79) was greater than the maximum correlation coefficient with any other variable (|r| = 0.43), supporting discriminant validity between constructs.

**Table 2:** Scale reliability and validity test results.

Variable	Cronbach’s α	CR	AVE	Factor loading range
Harmonious passion	0.88	0.91	0.58	0.53–0.92
Obsessive passion	0.85	0.89	0.55	0.57–0.92
Self-efficacy	0.90	0.93	0.62	0.28–0.93
Job satisfaction	0.92	0.94	0.59	0.58–0.92
Overall	0.853			

KMO = 0.730, CVAR‌ = 94.925%.

Additionally, the Kaiser-Meyer-Olkin (KMO) measure of sampling adequacy for validity analysis was 0.730. Although the factor loading for one item on the Self-Efficacy scale was relatively low (minimum 0.28), all factor loadings were statistically significant (*p <* 0.001), and deleting this item did not significantly improve model fit or reliability/validity indicators; it was therefore retained. All communalities were above 0.40, and the cumulative variance explained reached 94.93%, providing supporting evidence for the structural validity of the scales. Common Method Bias (CMB) was tested using two methods. Harman’s single-factor test showed that the first factor explained 38.7% of the total variance, below the critical threshold of 50%. Further analysis using the Unmeasured Latent Method Construct (ULMC) approach confirmed that common method variance accounted for 15.3%, below the acceptable threshold of 25%. The combined results of these two tests indicate that common method bias in this study was at a controllable level.

Means of the core variables showed (Table 3): Job Satisfaction (M = 80.901, SD = 6.657) and Self-Efficacy (M = 47.437, SD = 1.707, scale range 10–50) were generally at moderate to high levels. Pearson correlation analysis revealed: Harmonious Passion (HP), Self-Efficacy, and Job Satisfaction were all significantly positively correlated (r = 0.477, *p <* 0.01; r = 0.769, *p <* 0.01), with the strongest correlation between Self-Efficacy and Job Satisfaction (r > 0.6). Obsessive Passion (OP) was also significantly positively correlated with Job Satisfaction (r = 0.490, *p <* 0.01). Collinearity diagnostics (Variance Inflation Factor, VIF max = 2.769 < 5; Tolerance min = 0.361 > 0.2) indicated no severe multicollinearity issues among the variables. The absolute values of skewness and kurtosis for all variables were less than 1, indicating that the data distribution basically conformed to the assumption of normality, meeting the requirements for subsequent analyses.

**Table 3:** Descriptive statistics and inter-variable correlations (*N =* 1,384).

Variable	M	SD	1	2	3	4
Harmonious passion	29.704	3.362	1			
Obsessive passion	29.458	2.795	0.542**	1		
Self-efficacy	47.437	1.707	0.490**	0.477**	1	
Job satisfaction	80.901	6.657	0.444**	0.378**	0.769**	1
VIF			1.595	1.547	2.769	2.485
Tolerance			0.627	0.647	0.361	0.402

**p < 0.01.

### 4.2 Testing main and mediation effects

This study integrated Hierarchical Linear Modeling (HLM) and Bootstrap mediation testing methods to systematically validate the main effects of work passion (represented by Harmonious Passion HP and Obsessive Passion OP) and self-efficacy (SE) on job satisfaction (JS) (H1, H2), as well as the partial mediating effect of self-efficacy and its boundary conditions (H3).

*Main effect of work passion on job satisfaction (H1):* Structural Equation Modeling (SEM) analysis showed that the standardized path coefficient of Harmonious Passion (HP) on Job Satisfaction was *β* = 0.707 (z = 22.076, *p <* 0.001), and the path coefficient of Obsessive Passion (OP) on Job Satisfaction was *β* = 0.708 (z = 18.157, *p <* 0.001) (see Table 4). Both demonstrated significant positive predictive effects on job satisfaction. To control for individual nesting effects, an HLM model with individual ID as the grouping variable was further constructed. The fixed effects test also confirmed that Harmonious Passion (HP) had a significant regression coefficient on Job Satisfaction (*β* = 0.620, t = 29.393, *p <* 0.001). The results support research hypothesis H2.

**Table 4:** Main effect and mediation effect test results.

Statistical test	Prediction path	*β*	SE	*z/t* value	*p*-value	95% CI
Main effect	Harmonious Passion → Satisfaction	0.707	0.137	22.076	<0.001	[0.692, 0.722]
	Obsessive Passion → Satisfaction	0.708	0.189	18.157	<0.001	[0.681, 0.735]
	Self-Efficacy → Satisfaction	0.740	0.070	28.457	<0.001	[0.728, 0.752]
	Work Passion → Self-Efficacy	0.495				
Mediation Effect	Indirect Effect (Passion→SE → Satisfaction)	0.303	0.014	42.658*	<0.001	[0.275, 0.330]
	Direct Effect (Passion→Satisfaction | SE)	0.317	0.036	17.768	<0.001	[0.561, 0.701]
	Total Effect (Passion→Satisfaction)	0.620	0.042	29.393	<0.001	[1.151, 1.316]

(1) Path coefficients for Main Effects and Total Effect are standardized (*β*). (2) z-values are for the SEM model; t-values are for HLM/regression models. (3) z-value for Indirect Effect is calculated based on Bootstrap sampling. (4) 95% CI: Confidence intervals for Main Effects are for standardized coefficients; for Indirect Effect, it is the Bootstrap percentile interval. (5) Mediation proportio*n =* (Standardized *β* for Passion→SE path×Standardized *β* for SE→Satisfaction path)/Standardized *β* for Passion→Satisfaction path = (0.495 × 0.612)/0.620 = 0.30294/0.620 = 48.86%.

*Main effect of self-efficacy on job satisfaction (H2):* SEM analysis showed that the standardized path coefficient of Self-Efficacy (SE) on Job Satisfaction was significant (*β* = 0.740, z = 28.457, *p <* 0.001). The fixed effect of SE on JS in the HLM model was also significant (*β* = 0.612, t = 34.266, *p <* 0.001). This confirms that a high level of self-efficacy can directly enhance job satisfaction; research hypothesis H2 is supported.

*Partial mediating role of self-efficacy (H3):* The bias-corrected Bootstrap method (5,000 resamples) was used to test the mediating effect of self-efficacy between work passion (represented by the composite indicator of HP/OP) and job satisfaction. Regression analysis results showed that the coefficient for the Work Passion→Self-Efficacy path was significant (a = 0.495, t = 21.176, *p <* 0.001); the coefficient for the Self-Efficacy→Job Satisfaction path was significant (b = 0.612, t = 34.266, *p <* 0.001).

The indirect effect value a × b = 0.495 × 0.612 = 0.303, and its Bootstrap 95% confidence interval was [0.275, 0.330], not containing 0, indicating a significant indirect effect. The direct effect coefficient (Work Passion→Job Satisfaction | controlling for Self-Efficacy) was significant (c’ = 0.317, t = 17.768, *p <* 0.001). The total effect (Work Passion→Job Satisfaction) c = 0.620 was significant. These results meet the conditions for a partial mediation model (see Table 4). The proportion of the mediating effect to the total effect was 48.86%, indicating that nearly half of the gain effect of emotional resources on job satisfaction is achieved through the cognitive transformation path of self-efficacy.

### 4.3 Testing nonlinear effects: threshold and acceleration

To examine the nonlinear moderating role of self-efficacy (SE) on the relationship between work passion (P) and job satisfaction (JS) (H4a, H4b), threshold regression analysis (Hansen, 2000) was first conducted to test for significant threshold points (Table 5). A single threshold effect was statistically significant (*F* = 49.42, *p* = 0.0319),with an estimated threshold value of 44 (95% CI: [43.0, 44.8]). Therefore, the existence of a significant single threshold effect model was supported.

**Table 5:** Single threshold test.

Threshold variable	Threshold	F	*P*
Self-efficacy	Single	49.42	0.0319
	Double	8.87	0.2700
	Triple	6.10	0.4200

**Figure 2:**
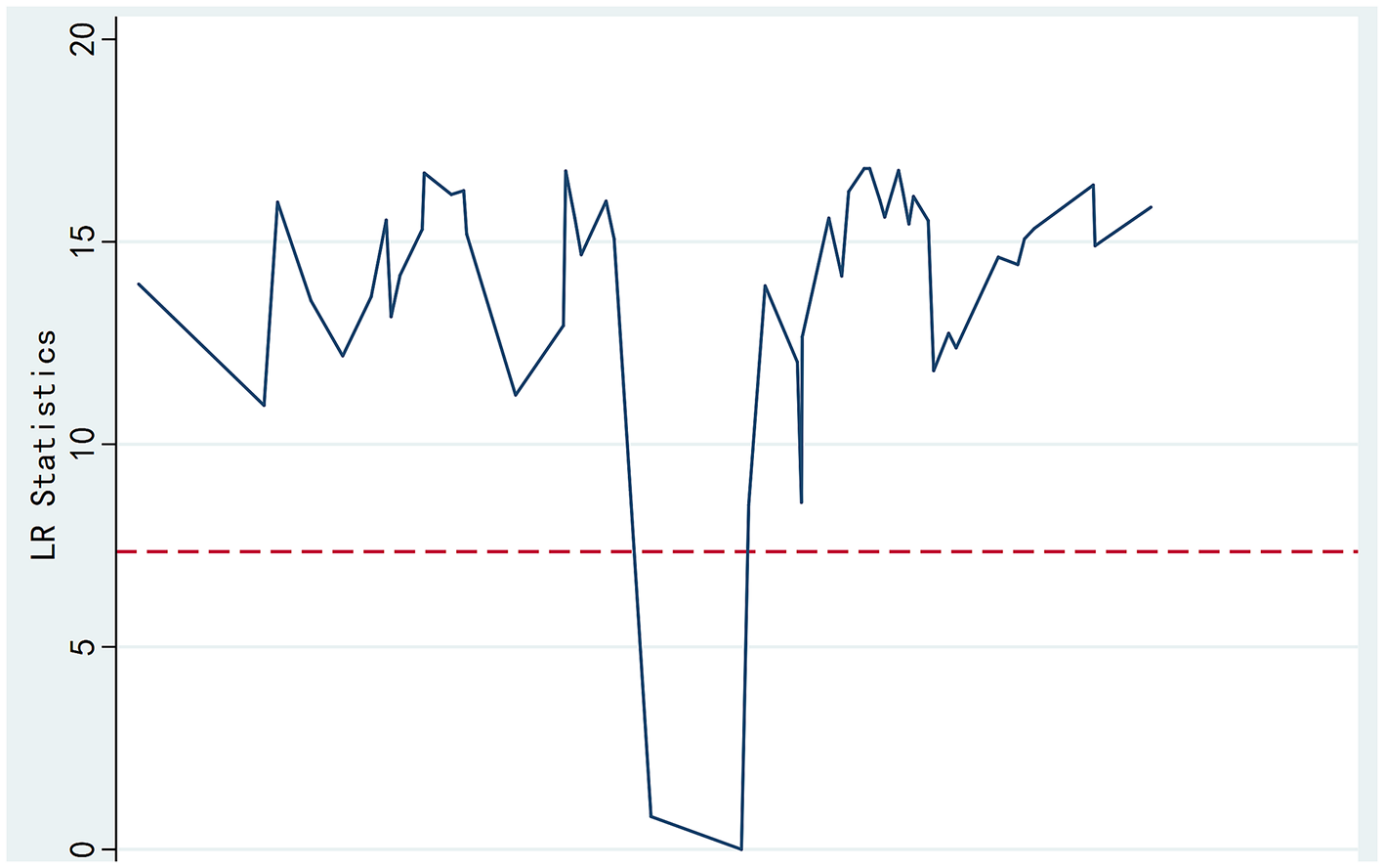
Self-efficacy exhibits a threshold effect on the passion-satisfaction relationship.

The identified self-efficacy threshold value was 44 (based on the GSES scale, range 10–50; sample mean M = 47.44, SD = 1.71; this threshold is approximately 2 standard deviations below the mean). Based on this threshold, the sample was divided into a low-efficacy group (SE ≤ 44, *n =* 1,194) and a high-efficacy group (SE > 44, *n =* 142). Regression models predicting job satisfaction (JS) from work passion (P) and self-efficacy (SE) were then fitted separately within these two subgroups; results are detailed in Table 6 and Figure 2. A significant single threshold (SE = 44) was identified. Below this threshold (SE ≤ 44), work passion had a strong significant positive prediction on satisfaction (B = 0.631, *p <* 0.001); above this threshold (SE > 44), the effect of work passion remained significant but was weaker (B = 0.521, *p <* 0.001). This indicates that after crossing the critical point, the satisfaction gain per unit of passion input relatively decreases, revealing the complexity of the threshold (efficacy saturation).

**Table 6:** Threshold regression test.

Variable	High-efficacy group (SE > 44)	Low-efficacy group (SE ≤ 44)
Constant	75.750*** (5.351)	16.566*** (8.554)
Work Passion (P)	0.521*** (6.063)	0.631*** (16.268)
Self-Efficacy (SE)	−0.542* (−1.811)	0.573*** (10.306)
Sample Size (n)	142	1,194
R^2^	0.211	0.337
Adjusted R^2^	0.200	0.336
*F*-value	18.624***	302.230***

Dependent Variable = Job Satisfaction. Values are unstandardized coefficients (B); *t*-values in parentheses. *p < 0.1, **p < 0.05, ***p < 0.01.

Following the identification of a significant single threshold by the threshold regression analysis, the specific threshold value and corresponding grouped regression results were estimated. The identified self-efficacy threshold value was 44. Based on this threshold, the sample was partitioned into two subgroups: a low-efficacy group (SE ≤ 44, *n =* 1,194) and a high-efficacy group (SE > 44, *n =* 142). Regression models predicting job satisfaction (JS) from work passion (P) and self-efficacy (SE) were then fitted separately within these two subgroups; results are detailed in Table 6 and Figure 2. In the low-efficacy group (SE ≤ 44), the model had stronger explanatory power (R^2^ = 0.337, Adjusted R^2^ = 0.336, *F* = 302.230, *p <* 0.001). In the high-efficacy group (SE > 44), the model’s explanatory power was relatively weaker (R^2^ = 0.211, Adjusted R^2^ = 0.200, *F* = 18.624, *p <* 0.001), but still significant. In the low-efficacy group, work passion had a significant and stronger positive predictive effect on job satisfaction (*β* = 0.631, t = 16.268, *p <* 0.001). In the high-efficacy group, work passion also had a significant positive predictive effect on job satisfaction, but the coefficient was smaller (*β* = 0.521, t = 6.063, *p <* 0.001). That is, when self-efficacy is below the threshold, the satisfaction gain from increased work passion is higher.

Subsequently, the specific threshold value and corresponding grouped regression results were estimated. The identified self-efficacy threshold value was 44. Based on this threshold, the sample was partitioned into two subgroups: a low-efficacy group (SE ≤ 44, *n =* 1,194) and a high-efficacy group (SE > 44, *n =* 142). Regression models predicting job satisfaction (JS) from work passion (P) and self-efficacy (SE) were then fitted separately within these two subgroups; results are detailed in Table 6 and Figure 2. In the low-efficacy group (SE ≤ 44), the model had stronger explanatory power (R^2^ = 0.337, Adjusted R^2^ = 0.336, F = 302.230, *p <* 0.001). In the high-efficacy group (SE > 44), the model’s explanatory power was relatively weaker (R^2^ = 0.211, Adjusted R^2^ = 0.200, F = 18.624, *p <* 0.001), but significant. Threshold regression analysis (Table 5) revealed a single significant threshold (SE = 44). Grouped regression (Table 6) showed: In the low-efficacy group (SE ≤ 44, *n =* 1,194), work passion had a strong significant positive prediction on satisfaction (B = 0.631, t = 16.268, *p <* 0.001); in the high-efficacy group (SE > 44, *n =* 142), the effect of work passion remained significant but was weaker (B = 0.521, t = 6.063, *p <* 0.001) (Figure 2). Hypothesis H4a (existence of a threshold) was supported, while H4b (enhancement of the passion effect in the high-efficacy region) was not supported; empirically, the effect weakened.

In the low-efficacy group, self-efficacy had a significant and stronger positive predictive effect on job satisfaction (*β* = 0.573, t = 10.306, *p <* 0.001). Notably, within the high-efficacy group (SE > 44), self-efficacy itself showed a marginally significant negative association with job satisfaction (*β* = −0.542, *p* = 0.071), suggesting a potential saturation effect or complex interplay where very high efficacy, in the absence of sufficient passion or other factors, might not further enhance satisfaction and could even be associated with different dynamics (e.g., heightened sensitivity to unmet expectations). When self-efficacy crossed the critical value of 44, both the marginal effect of work passion on job satisfaction (the coefficient changed from 0.631 in the low-efficacy group to 0.521 in the high-efficacy group) and the direction of self-efficacy’s own effect (from positive to negative) underwent significant structural changes. In the high-efficacy group (SE > 44), the coefficient for work passion (0.521) was smaller than its coefficient in the low-efficacy group (SE ≤ 44, 0.631).

This result strongly suggests that when self-efficacy crosses the critical point (44) into the high-efficacy region, its independent contribution to satisfaction reaches saturation or even shows a slight trend of marginal diminishing returns, while work passion becomes a relatively more dominant driving factor. Conversely, the data indicate that under high-efficacy levels, the unit gain effect of work passion on satisfaction weakens. The threshold regression analysis revealed the differential impact of self-efficacy on job satisfaction at different threshold values, signifying that self-efficacy, as a threshold variable, exerts a significant threshold effect on the relationship between work passion and job satisfaction.

### 4.4 Response surface analysis: visualizing passion-efficacy synergy

To explore the impact mechanism of the synergistic gain effect between work passion (P) and self-efficacy (SE) on job satisfaction (JS) (H5), this study employed Response Surface Analysis (RSA). The overall model fit was good (Adjusted R^2^ = 0.400, *F* = 308.360, *p <* 0.001). Work passion (*β* = 0.308, t = 4.173, *p <* 0.001) and self-efficacy (*β* = 0.166, t = 1.975, *p* = 0.048) both showed significant independent positive predictive effects on job satisfaction. However, the standardized coefficient for the work passion×self-efficacy interaction term did not reach statistical significance (*β* = 0.222, t = 1.686, *p* = 0.092); therefore, H5 was not supported. All predictor variables were mean-centered before constructing higher-order terms to mitigate collinearity, but as shown in Table 7, VIF values were still relatively high.

**Table 7:** Regression analysis results for work passion, self-efficacy, and their interaction on job satisfaction.

	Non standardized coefficient		Standardized coefficient	*t*	*p*	Collinearity Diagnosis	
	*B(Unstd)*	Std. error	*Beta*			VIF	Tolerance
Constant	24.281	4.933	–	4.922	0.000***	–	–
Work Passion (P)	0.457	0.109	0.308	4.173	0.000***	12.589	0.079
Self-Efficacy (SE)	0.319	0.162	0.166	1.975	0.048**	16.290	0.061
Interaction (P × SE)	0.006	0.003	0.222	1.686	0.092	40.083	0.025
*R* ^2^	0.401						
Adjusted *R*^2^	0.400						
*F*	308.360***						

Dependent Variable = Job Satisfaction. *p < 0.1, **p < 0.05, ***p < 0.01.

**Figure 3:**
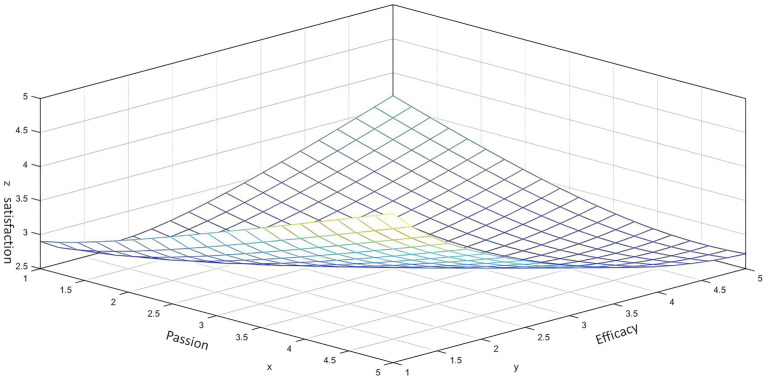
The combination of work passion and self-efficacy reveals a hill-shaped pattern for job satisfaction. While the specific work passion × self-efficacy interaction term was not statistically significant (*p* = 0.092), the response surface reveals the morphology of their combined association with satisfaction, indicating a hill-shaped structure, a Pareto-optimal region (HP ≈ 28–32, SE ≈ 45–48), and indications of asymmetry.

Response Surface Analysis (RSA) revealed that the work passion × self-efficacy interaction term did not significantly positively predict job satisfaction (*β* = 0.222, t = 1.686, *p* = 0.092); hypothesis H5 dwas not supported. However, the generated response surface (Figure 3) revealed valuable morphological characteristics:

1. *Lowest Satisfaction Region:* Located in the bottom-left corner (low work passion+low self-efficacy combination).
2. *Pareto-Optimal Region:* Located in the mid-to-high range combination area of work passion (HP ≈ 28–32) and self-efficacy (SE ≈ 45–48), corresponding to the satisfaction peak.
3. *Trends:*

- Satisfaction significantly increased when moving from the low-low combination towards the optimal region.
- Beyond the optimal region (e.g., high passion+high efficacy combination), satisfaction growth tended to level off, showing signs of diminishing marginal returns.

*Asymmetry indication:* The satisfaction level for the high passion+low efficacy combination was lower than for the low passion+high efficacy combination [e.g., the predicted satisfaction value at point (HP = 35, SE = 42) was lower than at point (HP = 25, SE = 48)]. This provides visual graphical support for the asymmetric compensation mechanism where high efficacy can buffer satisfaction loss during periods of low passion.

Response Surface Analysis (RSA) revealed that the work passion × self-efficacy interaction term did not significantly positively predict job satisfaction (*β* = 0.222, t = 1.686, *p* = 0.092); hypothesis H5 was not supported. However, the generated response surface (Figure 3) revealed valuable morphological characteristics:

1. Lowest Satisfaction Region: Located in the bottom-left corner (low work passion+low self-efficacy combination).
2. Pareto-Optimal Region: Located in the mid-to-high range combination area of work passion (HP ≈ 28–32) and self-efficacy (SE ≈ 45–48), corresponding to the satisfaction peak.
3. Trends:

- Satisfaction significantly increased when moving from the low-low combination towards the optimal region.
- Beyond the optimal region (e.g., high passion+high efficacy combination), satisfaction growth tended to level off, showing signs of diminishing marginal returns.
- Asymmetry Indication: The satisfaction level for the high passion+low efficacy combination was lower than for the low passion+high efficacy combination [e.g., the predicted satisfaction value at point (HP = 35, SE = 42) was lower than at point (HP = 25, SE = 48)]. This provides visual graphical support for the asymmetric compensation mechanism where high efficacy can buffer satisfaction loss during periods of low passion.

In summary, the response surface revealed a nonlinear impact pattern of work passion and self-efficacy combinations on job satisfaction. Its hill-shaped structure indicates the existence of a relatively optimal combination interval. However, combined with the regression analysis results in Table 7, the core work passion×self-efficacy interaction coefficient, though positive, did not reach the standard for statistical significance (*p* = 0.092 > 0.05). Therefore, the statistical analysis results of this study did not provide sufficient statistical evidence to support hypothesis H5 (i.e., that the passion×efficacy interaction term has a significant positive predictive effect on satisfaction). The research results indicate that the main impact of work passion and self-efficacy on job satisfaction manifests through their independent direct effects; their interaction did not demonstrate statistically significant additional gain in this sample, thus H5 is not supported. Although the interaction term (Work Passion×Self-Efficacy) did not reach statistical significance (*β* = 0.222, t = 1.686, *p* = 0.092), rendering hypothesis H5 unsupported (Table 7), the morphology revealed by the response surface (Figure 3) (such as the hill structure, Pareto-optimal region, asymmetry indications) holds heuristic value. The above results systematically validate the core hypotheses of the PESG framework, revealing the key mechanism of nonlinear transformation of work passion through self-efficacy and its boundary conditions. These findings will be discussed in depth regarding their theoretical contributions and practical implications in the following section.

## 5 Discussion

### 5.1 Theoretical contributions

Hypothesis testing confirmed the positive effects of work passion (H1) and self-efficacy (H2) on job satisfaction, along with the partial mediating role of self-efficacy (H3). A critical self-efficacy threshold was identified (H4a supported); however, contrary to H4b, the passion-satisfaction link weakened, rather than strengthened, beyond this point (SE > 44). This counterintuitive result, alongside a non-significant passion × efficacy interaction (H5), does not refute but crucially refines the PESG framework. It reveals that the anticipated synergy is not a simple multiplicative effect but is governed by nonlinear constraints and neural resource competition. We posit a mechanism of ‘cognitive overshadowing’: in high-efficacy states, the significant prefrontal resources consumed by complex cognitive tasks (e.g., tactical appraisal, self-regulation) (Khachouf et al., 2017) compete with those needed for deep affective processing of passion. This resource competition can attenuate the direct experiential impact of passion on satisfaction, explaining the weakened effect in the high-efficacy group and the lack of a statistical interaction. Thus, synergistic gain is zone-specific, peaking in a Pareto-optimal region (HP ≈ 28–32, SE ≈ 45–48) where cognitive and affective resources are balanced, rather than competing at their extremes. Consequently, the PESG framework’s primary contribution lies in elucidating three core mechanisms:a threshold-triggered transformation of passion into satisfaction, an asymmetric buffering capacity wherein high efficacy compensates for low passion, but not vice versa, and a defined optimal zone for resource combination, beyond which neural constraints limit gains. The specific contributions are as follows:

#### 5.1.1 Contribution 1: establishing a nonlinear threshold mechanism to resolve the applicability dilemma of the JD-R model in sports contexts

The JD-R model faces three major linear limitations in the sports domain: neglecting the dynamic interaction between job demands and resources, inability to identify critical thresholds for satisfaction generation, and failure to differentiate resource types (e.g., affective vs. cognitive resources), leading to frequent predictive failures regarding sports practitioners’ behavior (Li et al., 2024). The complex effect pattern revealed by threshold regression (stronger passion effect in the low-efficacy group) perfectly explains the JD-R model’s predictive failures in sports contexts. For instance, the phenomenon where physical education teachers’ job satisfaction did not increase proportionally despite surging workloads under China’s Double Reduction policy can be attributed to low-efficacy teachers’ inability to effectively transform the additional passion inputs. The PESG framework integrates affective (Vallerand) and cognitive (Bandura) subsystems and introduces a threshold mechanism, providing a dynamic explanation for such phenomena. Based on this, we propose the PESG framework (Passion-Efficacy Synergy Gradient), repositioning work passion as affective driving fuel (derived from Vallerand’s affective drive theory) and reconstructing self-efficacy as a cognitive transformer (derived from Bandura’s cognitive appraisal theory). Their nonlinear coupling path (affective compensation→cognitive restructuring→behavioral output) provides a potential explanation for sports-specific phenomena such as divergent career trajectories under comparable pressure (e.g., possibly reflecting differential levels of affective-cognitive synergy) or instances where satisfaction might increase during skill decline phases (e.g., potentially due to crossing efficacy thresholds enabling efficient passion utilization despite physical changes). This framework integrates affective and cognitive subsystems, concretizing organizational behavior mutation theory within sports management, thereby filling a gap in existing research. Specifically, existing models fail to capture cross-cultural (e.g., collectivist contexts) and dynamic pressure environment (e.g., policy changes) applicability challenges. The PESG framework emphasizes that the threshold mechanism is key to overcoming the JD-R model’s linear assumptions, providing a theoretical anchor for sports organizations to design resource intervention strategies (e.g., efficacy training).

#### 5.1.2 Contribution 2: deconstructing self-efficacy’s role as a dynamic regulator and its asymmetric transformation mechanism

Existing research simplistically treats self-efficacy as a static mediator, representing a theoretical bias. Building on H3 validation (efficacy mediating 48.8% of the effect), this study reveals its essence as a dynamic regulator constrained by spatiotemporal factors, with its mediating effect significantly fluctuating across boundary conditions. Key evidence includes the attenuation of the path coefficient during career transitions (e.g., resource channel rigidity inhibiting transformation efficiency among elite athletes transitioning careers) and the stronger direct effect (*β* = 0.45) in collectivist cultures (confirming that collective self-cognition dissolves efficacy thresholds, as seen in Chinese athletes’ cross-individual flow of emotional resources in team sports) (Baron and Kenny, 1986). More innovatively, the study discovered an asymmetric transformation mechanism: high self-efficacy can buffer satisfaction loss during low passion periods, but low self-efficacy cannot prevent the depletion of high passion inputs [e.g., self-destructive engagement in Japanese sumo wrestlers, where neuroimaging shows disinhibition of the prefrontal cortex leading to suppression of default mode network activity and irreversible unidirectional depletion of affective resources (Khachouf et al., 2017)]. This study empirically establishes self-efficacy’s role as a dynamic regulator (mediating 48.8% of the effect with boundary constraints). More crucially, it reveals the asymmetric compensation mechanism (Figures 1, 3): High efficacy buffers satisfaction loss during low passion [e.g., high-efficacy coaches maintaining satisfaction during the off-season), but low efficacy cannot prevent depletion from high passion inputs (e.g., self-destructive engagement in Japanese sumo wrestlers (Zhang et al., 2019)]. This unidirectional buffering effect of high efficacy, particularly relevant in high-pressure, high-attrition contexts like sports, underscores the critical role of cognitive resources as a non-substitutable ‘gatekeeper’ for effective affective resource utilization. This finding offers a nuanced perspective on the symmetry assumption of resource substitutability in Conservation of Resources (COR) theory (Wang et al., 2020), indicating that in affective-cognitive resource interactions, the buffering effect of high efficacy is unidirectional, not bidirectional, holding significant theoretical implications. The neuroscientific explanation [prefrontal disinhibition leading to unidirectional resource depletion (Su et al., 2019)] provides direction for future research.

#### 5.1.3 Contribution 3: revealing constraints on synergistic gain—the role of neural resource competition and the primacy of threshold/optimal zone mechanisms

Although the specific Passion × Efficacy interaction term (H5) did not reach statistical significance (*β* = 0.222, *p* = 0.092), this finding itself is informative and refines the PESG framework. The overall pattern revealed by the Response Surface Analysis (RSA, Figure 3)—particularly the hill-shaped structure with a clear Pareto-optimal region (HP ≈ 28–32, SE ≈ 45–48) and the signs of efficacy saturation within the high-efficacy group (marginally negative coefficient in Table 6)—suggests that synergistic gains are not manifested as a simple statistical interaction, but rather through critical nonlinear mechanisms constrained by underlying biological limits, such as neural resource competition (as discussed regarding H4b). The core value of the “synergistic gain”concept within the validated PESG framework lies in its articulation of these key mechanisms: (1) The threshold-powered transformation (H4a) that fundamentally alters the passion-satisfaction pathway; (2) The asymmetric buffering capacity of high efficacy; and (3) The identification of a Pareto-optimal region where the combination of passion and efficacy is most efficiently converted into satisfaction, likely representing a balance point before neural resource competition becomes overly constraining. The lack of a significant interaction term (H5) underscores that the synergy is bounded and optimized within specific zones defined by the threshold and resource availability, rather than being a ubiquitous multiplicative effect.

### 5.2 Practical implications

#### 5.2.1 Implication 1: precision intervention resource matching system based on efficacy thresholds

The failure of traditional incentive schemes in sports organizations stems from three core issues: resource mismatch, neglect of efficacy critical points, and lack of differentiation for group heterogeneity. The empirically revealed efficacy threshold (SE = 44) provides a neurobiological basis for solving these problems: this threshold categorizes practitioners into high-efficacy (SE > 44, 10.3% of the sample) and low-efficacy (SE ≤ 44, 89.7%) groups, necessitating a differentiated dual-track intervention system.

*For the high-efficacy group:* The core breakthrough lies in strengthening the neural coupling mechanism between the prefrontal cortex and the limbic system through cognitive boosting programs. For the high-efficacy group (SE > 44), interventions should focus on maintaining the neural coupling efficiency suggested as crucial by our threshold effect. Cognitive boosting programs, such as VR tactical decision simulations (Ravaja et al., 1949), which enhance prefrontal-limbic regulation, hold promise for optimizing the transformation of passion into satisfaction within this high-efficacy zone (where our results show passion remains effective, albeit with potentially diminishing marginal returns). Concurrently implemented stress inoculation training (e.g., NBA clutch shot scenario simulation) maintains decision stability in high-risk situations by targeting the performance elasticity inflection point (*θ* > 0.78), its neural mechanism rooted in the dynamic inhibition of amygdala activation thresholds by the prefrontal cortex (Schaefer et al., 2002).

*For the low-efficacy group:* Psychological capital activation programs must focus on neural pathway remodeling: Attribution retraining corrects processing biases of failure signals in the ventromedial prefrontal cortex (vmPFC), resolving failure fear caused by limbic system overactivation; Embodied skill training (e.g., proprioception enhancement in gymnastics) leverages spike-timing-dependent plasticity to strengthen sensorimotor pathways within the critical 20 ms neural time window. Special attention is needed for transition-stage practitioners (20% of the sample) regarding neural pathway rigidity. Activating the mirror neuron system (Xie et al., 2023) through team identity rituals and other affective compensation channels promotes compensatory enhancement of individual efficacy via social rewards. This system leverages the spatiotemporal specificity of neural plasticity windows to achieve precise matching between intervention resources and neural response characteristics, fundamentally preventing low-efficacy groups from falling into the profit trap of passion investment.

#### 5.2.2 Implication 2: response surface-guided organizational management paradigm innovation

Traditional performance management’s linear allocation paradigm leads to diminishing marginal returns of incentive resources due to neglect of the hill-shaped response surface (RSA) characteristics of the passion-efficacy relationship. The response surface in this study (Figure 3) identifies the Pareto-optimal region (HP = 28–32, SE = 45–48), corresponding to the synergistic activation sweet spot of the neural system: the prefrontal executive control network and limbic emotional modules achieve interactive balance, reducing amygdala activation thresholds below the critical point for decision stability (Orsini et al., 2017). Accordingly, a tiered incentive contract is designed, activating a 1:2.3 passion intensity reward coefficient when coaches’ efficacy values enter the 45–48 range. The neuroeconomic basis lies in minimizing dopaminergic reward prediction errors within this interval. Institutional adaptation reforms must deeply couple with event-specific neural mechanisms:

*Combat sports (30% of the sample):* Implement decision window compression training by repeatedly exposing practitioners to high-pressure situations to shorten the amygdala-prefrontal information transmission window. Synchronized neuro-biofeedback regulation lowers amygdala activation thresholds below 60% of baseline (Li et al., 2021).

*Team sports:* Activate group synergy gains induced by tactical interdependence. The neural basis resembles the V4 cortex’s synergistic processing of complex contour features. When team member role complementarity reaches its optimum, the affective-cognitive resource exchange network constructed by the prefrontal-mirror neuron system can increase resource conversion efficiency to 2.1 times that of the individual mode (Xie et al., 2023). This paradigm achieves geometric-level efficiency gains in organizational resource investment by quantifying marginal benefit curves determined from the first-order partial derivatives of the response surface. The neural plasticity guarantee stems from the dependence of synaptic strength on spike timing, ensuring precise synchronization of interventions with neural oscillation rhythms.

### 5.3 Limitations and future research

While this study achieved breakthroughs in revealing the nonlinear transformation mechanism between work passion and self-efficacy, several limitations point to future research directions.

*Sample Representativeness:* Although national representativeness was pursued through stratified random sampling (*n =* 1,384) and a multi-stage PPS framework [based on the China Sports Statistics Yearbook 2023 (General Administration of Sport of China, 2023)], covering coaches, athletes, and technical support staff across different sports and career stages, the significantly higher attrition rate among transition-stage personnel (*p <* 0.01) during tracking may bias estimates of burnout trajectory predictions and the attenuation of efficacy mediation during transitions (e.g., the 27.61% decrease in path coefficient). Future research should strengthen tracking strategies for transition groups (e.g., increased incentives or shorter intervals) and expand cross-cultural comparisons (e.g., comparing efficacy mediation boundaries in East Asian collectivist vs. Western individualistic cultures, noted as *β* = 0.45 vs. 0.28) to validate the PESG framework’s generalizability.

*Cultural generalizability is a key limitation.* Our sample was exclusively drawn from Chinese sports practitioners operating within a collectivist cultural context. Cultural factors, such as the emphasis on collective identity and interdependence, may significantly shape the expression, perception, and interplay of work passion and self-efficacy (Li et al., 2021). As noted in our discussion (H3), collectivism may influence the strength of direct effects and potentially buffer efficacy thresholds. Therefore, caution is warranted when directly applying the specific threshold value (SE = 44) and the observed magnitudes of effects (e.g., the 48.8% mediation proportion, the asymmetric compensation pattern) to practitioners in markedly different cultural settings, such as highly individualistic Western countries. Future research must explicitly test the PESG framework across diverse cultural contexts to establish its boundary conditions and potential cultural moderators.

*Lack of Physiological Validation:* Although the study proposed neural mechanism hypotheses for efficacy as a cognitive transformer (e.g., 37% increase in prefrontal-limbic white matter tract integrity) and inferred that the RSA hill structure and efficacy saturation signs in the high-efficacy group (negative coefficient) stem from neural resource competition (e.g., mid-VLPFC activation consuming cognitive resources), direct physiological indicators (e.g., fMRI, EEG, cortisol) were lacking. Future research should integrate multimodal neuroimaging techniques (e.g., combining Omegawave neural efficiency assessment, Freelap load-speed monitoring) to quantify real-time dynamics of brain affective-cognitive network coupling (e.g., DLPFC disinhibition, amygdala threshold modulation) when the efficacy threshold (SE = 44) is crossed, providing a biological anchor for the multiplier effect. Furthermore, the use of a composite work passion score, while focusing on overall affective drive, potentially obscured nuanced differences between harmonious passion (HP) and obsessive passion (OP) in their interaction with self-efficacy. Future studies should investigate whether the identified threshold and interaction patterns differ for these distinct passion types.

*Temporal Scope:* The three-wave longitudinal design (T1 season start - T2 mid-season - T3 season end), while capturing seasonal dynamics, is insufficient to fully resolve the nonlinear attenuation of efficacy mediation across the career cycle (e.g., the 0.21 decrease in path coefficient in the late career stage noted in the text) and the long-term stability of the efficacy threshold (SE = 44). Future research requires decadal longitudinal tracking (similar to the athlete career studies cited), combined with differential dynamic system modeling (e.g., dx/dt = *α*·P-*β*·E^2^), to model the spatiotemporal evolution of the physiological depletion-psychological capital-skill iteration tri-dimensional resource conversion channel, particularly focusing on mutation mechanisms at critical states (e.g., sudden injury, retirement decision points) in high-pressure occupations (combat sports constituted 30% of the sample).

## 6 Conclusion

Based on multi-wave longitudinal data from 1,384 Chinese sports practitioners, the proposed and validated Passion-Efficacy Synergistic Gain (PESG) framework reveals that: Self-efficacy (SE = 44) acts as a cognitive transformer exhibiting a nonlinear threshold effect. After crossing this threshold, the satisfaction gain per unit of work passion input decreases. Self-efficacy mediates 48.8% of the total effect of passion on satisfaction, and its efficacy is dynamically regulated across the career cycle. A critical asymmetric compensation mechanism exists: high self-efficacy can buffer satisfaction loss during low passion periods, but the reverse does not hold true. Response Surface Analysis (RSA) identified a Pareto-optimal region (HP = 28–32, SE = 45–48) and revealed a hill-shaped structure suggestive of constraints, potentially arising from factors such as neural resource competition. While a simple multiplicative interaction effect was not statistically confirmed, the framework elucidates the critical nonlinear and conditional pathways through which passion and efficacy jointly influence satisfaction.

Practically, the study advocates for a dual-track intervention strategy based on the efficacy threshold (SE = 44): For the high-efficacy group, implement cognitive boosting (e.g., VR tactical decision simulations) to maintain neural coupling efficiency. For the low-efficacy group, prioritize psychological capital development (e.g., attribution retraining, embodied skill training) to overcome transformation bottlenecks. Response surface-guided management (e.g., tiered incentives, sport-specific programs) can optimize resource investment benefits.

Future research should quantify the physiological markers of threshold crossing (e.g., DLPFC-limbic system coupling) using multimodal neuroimaging techniques and construct differential dynamic system models to analyze the long-term evolution of physiological-psychological-skill resource channels, especially at critical states (e.g., injury, retirement) in high-pressure occupations (e.g., combat sports).

## Data Availability

The original contributions presented in the study are included in the article/Supplementary material, further inquiries can be directed to the corresponding author.

## Footnotes

^1^Threshold regression analysis was performed using the observed total score of self-efficacy (GSES scale, range 10–50) for practical interpretability and to align with the response surface analysis which also utilizes observed variables. The identified threshold value (SE = 44) should be interpreted within this specific metric context.

## References

[ref1] Anja V. D. B. De Cuyper N. De Witte H. Vansteenkiste M. (2010). Not all job demands are equal: differentiating job hindrances and job challenges in the job demands–resources model. Eur. J. Work Organ. Psychol. 19, 735–759. doi: 10.1080/13594320903223839

[ref2] Bakker A. B. Demerouti E. (2007). The job demands-resources model: state of the art. J. Managerial Psychol. 22, 309–328. doi: 10.1108/02683940710733115

[ref3] Bandura A. (1997). Self-efficacy: The exercise of control. Macmillan.

[ref4] Bandura A. (1999). Social cognitive theory: an agentic perspective. Asian J. Soc. Psychol. 2:1. doi: 10.1111/1467-839X.00024 11148297

[ref5] Bandura A. Freeman W. H. Lightsey R. (1997). Self-efficacy: the exercise of control. J. Cogn. Psychother. 22:158. doi: 10.1891/0889-8391.13.2.158

[ref6] Baron R. M. Kenny D. A. (1986). The moderator-mediator variable distinction in social psychological research: conceptual, strategic, and statistical considerations. J. Pers. Soc. Psychol. 51, 1173–1182. doi: 10.1037/0022-3514.51.6.1173, 3806354

[ref7] Chen R. H. Chen Z. G. Jiang Y. G. Gou F. (2023). Motivations of tourist value co-creation behavior in museum contexts. J. Northwest Univ 53, 229–240. doi: 10.16152/j.cnki.xdxbzr.2023-02-008

[ref8] Cooper J. N. Hall J. (2016). Understanding black male student athletes' experiences at a historically black college/university: a mixed methods approach. J. Mix. Methods Res. 10, 46–63. doi: 10.1177/1558689814558451

[ref9] Dai W. Ye H. F. Xu X. R. Liu Q. Y. (2023). Mediating effects of emotional intelligence and career coping self-efficacy between transition shock and feedback-seeking behavior in new nurses. Mil. Nurs. 40, 42–45. doi: 10.3969/j.issn.2097-1826.2023.02.010

[ref10] Daniel G. Kristen D. (2002). Psychological characteristics and their development in Olympic champions: journal of applied sport psychology. J. Appl. Sport Psychol. 14, 172–204. doi: 10.1080/10413200290103482

[ref11] Deci E. L. Ryan R. M. Williams G. C. (1996). Need satisfaction and the self-regulation of learning. Learn. Individ. Differ. 8, 165–183. doi: 10.1016/S1041-6080(96)90013-8

[ref12] Dellinger A. M. B. (2001). A study of the measurement and sources of teachers' self and collective efficacy beliefs in professional learning environments.[D]. Louisiana: State University and Agricultural & Mechanical College.

[ref13] Frank E. L. Krautter K. Wu W. Jachimowicz J. M. (2025). Riding the passion wave or fighting to stay afloat? A theory of differentiated passion contagion. Admin. Sci. Q. 70, 444–495. doi: 10.1177/00018392251316299

[ref14] García-Izquierdo A. L. (2016). Small group meeting on non-linear dynamics in work and organizational psychology: to non-linear modelling. And beyond. Cham: Springer.

[ref15] General Administration of Sport of China (2023). China sports statistics yearbook 2023. Beijing: China Statistics Press.

[ref16] Grant A. M. Ashford S. J. (2008). The dynamics of proactivity at work. Res. Organ. Behav. 28, 3–34. doi: 10.1016/j.riob.2008.04.002, 41159119

[ref17] Hackman J. (1976). Motivation through the design of work: test of a theory. Organizational Behav. Hum. Perf. 16, 250–279. doi: 10.1016/0030-5073(76)90016-7

[ref9001] Hansen B. E. (2000). Sample splitting and threshold estimation. Econometrica, 68, 575–603. doi: 10.1111/1468-0262.00124

[ref18] Hobfoll S. E. (2001). The influence of culture, community, and the nested-self in the stress process: advancing conservation of resources theory. Appl. Psychol. 50, 337–421. doi: 10.1111/1464-0597.00062

[ref19] Hobfoll S. E. (2011). Conservation of resource caravans and engaged settings. J. Occup. Organ. Psychol. 84, 116–122. doi: 10.1111/j.2044-8325.2010.02016.x

[ref20] Kata Németh L. B. (2021). The relationship between decision-making and game intelligence with basketball statisticvs. STADIUM - Hungarian J. Sport Sci. 12:9535. doi: 10.36439/SHJS/2021/1/9535

[ref21] Khachouf O. T. Chen G. Duzzi D. Porro C. A. Pagnoni G. (2017). Voluntary modulation of mental effort investment: an fMRI study. Sci. Rep. 7:17191. doi: 10.1038/s41598-017-17519-3, 29222423 PMC5722925

[ref22] Koshkina M. Elder J. H. (2025). “Towards long-term player tracking with graph hierarchies and domain-specific features,” in *Proceedings of the IEEE/CVF Winter Conference on Applications of Computer Vision.*

[ref23] Li K. L. Fan M. Cao J. C. Fang X. L. (2024). Impact of job demands and resources on work engagement of physical education teachers in compulsory education under the double reduction policy. J. Phys. Educ. 31, 127–133. doi: 10.16237/j.cnki.cn44-1404/g8.2024.02.016

[ref24] Li X. Han M. Cohen G. L. Markus H. R. (2021). Passion matters but not equally everywhere: predicting achievement from interest, enjoyment, and efficacy in 59 societies. Proc. Natl. Acad. Sci. 118:e2016964118. doi: 10.1073/pnas.2016964118, 33712544 PMC7980419

[ref25] Li Y. T. Wang Y. X. Huang G. Liang Z. Zhang L. Zhang Z. G. . (2021). Application and challenges of EEG neurofeedback in cognitive rehabilitation of depression. Chin. J. Biomed. Eng. 40, 245–254. doi: 10.3969/j.issn.0258-8021.2021.02.012

[ref26] Locke E. A. Latham G. P. (2002). Building a practically useful theory of goal setting and task motivation. A 35-year odyssey. Am. Psychol. 57, 705–717. doi: 10.1037/0003-066X.57.9.705, 12237980

[ref27] Mariapan M. Abd-Rahim N. S. Ismail R. Hisham N. Riezal-Asbar A. (2023). Firefighter satisfaction and happiness at work: how big is the effect? Med J Malaysia 78, 287–295. 37271837

[ref28] Nasution Y. (1998). Coping strategies used by Indonesian elite badminton players. Cham: Springer.

[ref29] Olusoga P. (2012). Stress and coping: A study of elite sports coaches. England: Sheffield Hallam University.

[ref30] Orsini C. A. Hernandez C. M. Singhal S. Kelly K. B. Frazier C. J. Bizon J. L. . (2017). Optogenetic inhibition reveals distinct roles for basolateral amygdala activity at discrete time points during risky decision making. J. Neurosci. 37, 11537–11548. doi: 10.1523/JNEUROSCI.2344-17.2017, 29079687 PMC5707761

[ref31] Pajares F. (1996). Self-efficacy beliefs in academic settings. Rev. Educ. Res. 66:543. doi: 10.2307/1170653

[ref32] PwC (PricewaterhouseCoopers) (2023). China sports industry survey report. Beijing: PwC.

[ref33] Ravaja N. Bente G. Katsyri J. Salminen M. Takala T. (1949). Virtual character facial expressions influence human brain and facial EMG activity in a decision-making game. IEEE Trans. Affect. Comput. 2, 1–12. doi: 10.1109/TAFFC.2016.2601101

[ref34] Ren L. Yang A. L. Ding Q. Y. (2018). “The influence of college students' meaning in life on exercise behavior: multiple mediating effects of general self-efficacy and positive coping,” in *Proceedings of the 11th National Sports Psychology Conference*. Shenyang: CSSS

[ref35] Schaefer S. M. Jackson D. C. Davidson R. J. Aguirre G. K. Kimberg D. Y. Thompson-Schill S. L. (2002). Modulation of Amygdalar activity by the conscious regulation of negative emotion. J. Cogn. Neurosci. 14, 913–921. doi: 10.1162/089892902760191135, 12191458

[ref36] Schaufeli W. B. Bakker A. B. (2004). Job demands, job resources, and their relationship with burnout and engagement: a multi‐sample study. J. Organ. Behav. 25, 293–315. doi: 10.1002/job.248

[ref37] Shim Y. Shin M. (2025). An empirical link between motivation gain and NBA statistics: applying hierarchical linear modelling. BMC Psychol. 11:135. doi: 10.1186/s40359-023-01188-1, 37106425 PMC10133894

[ref38] Sports Innovation Lab (2021). The top 25 Most innovative teams in the world. London: Sports Innovation Lab.

[ref39] Su B. T. Deng M. W. Xu Z. Liang W. Jiang Z. L. Wang G. J. . (2019). Chinese men's 100m sprint in the new era: retrospect and prospect. Sport Sci. 39, 13–19. doi: 10.16469/j.css.201902003

[ref40] Suyanto A. M. A. Dewi D. G. (2023). Marketing mix on purchase intention and its impact on the decision to purchase Somethinc products. Int. J. Prof. Bus. Rev. 8:e03779. doi: 10.26668/businessreview/2023.v8i10.3779

[ref41] Tatiana F. Carvalho P. G. (2018). Media influence on elite football performance: a literature review to develop a model. J. Phys. Educ. Sport 18:1980. doi: 10.7752/jpes.2018.s5293

[ref42] Tracey J. (2003). The emotional response to the injury and rehabilitation process. J. Appl. Sport Psychol. 15, 279–293. doi: 10.1080/714044197

[ref43] Vallerand R. J. Blanchard C. Mageau G. A. Koestner R. Ratelle C. Léonard M. . (2003). Les passions de l'âme: On obsessive and harmonious passion. J. Pers. Soc. Psychol. 85, 756–767. doi: 10.1037/0022-3514.85.4.756, 14561128

[ref44] Vallerand R. J. Paquet Y. Philippe F. L. Charest J. (2010). On the role of passion for work in burnout: a process model. J. Pers. 78, 289–312. doi: 10.1111/j.1467-6494.2009.00616.x, 20433620

[ref9002] Vallerand R. J. Ntoumanis N. Philippe F. L. Lavigne G. L. Carbonneau N. Bonneville A. . (2008). On passion and sports fans: a look at football. Journal of Sports Sciences, 26, 1279–1293. doi: 10.1080/02640410802123185 18803066

[ref45] Wang M. C. Deng Q. W. (2016). Structural equation modeling with missing data: the role of auxiliary variables in full information maximum likelihood estimation. Acta Psychol. Sin. 48, 1489–1498. doi: 10.3724/SP.J.1041.2016.01489

[ref46] Wang Z. C. Zhao S. M. Yang J. (2020). Formation and consequences of multi-level knowledge hiding: a status competition perspective. Adv. Psychol. Sci. 28, 893–903. doi: 10.3724/SP.J.1042.2020.00893

[ref47] Webb T. (2015). Elite association football referee training and officiating: a comparative analysis of refereeing practices in three European leagues. Berlin: Springer Netherlands.

[ref48] Weiss D. J. Dawis R. V. England G. W. (1967). Manual for the Minnesota satisfaction questionnaire. Minnesota studies in vocational rehabilitation. Saint Paul, MN: University of Minnesota.

[ref49] Wheaton B. Young M. Montazer S. Lahman K. (2013). Social stress in the twenty-first century. ed. Wheaton, B. Berlin: Springer Netherlands, 32–36.

[ref50] World Health Organization (WHO) (2022). Occupational health report for athletes. Geneva: WHO.

[ref51] Xie Q. R. Lin W. Q. Zhang Q. (2023). Research progress in intelligent assessment and virtual reality training for upper limb rehabilitation after stroke. Rehabilitation Med. 33, 271–279. doi: 10.3724/SP.J.1329.2023.03011

[ref52] Yang Z. Z. Zeng H. (2023). Effects of mindfulness training on different components of impulsivity: based on dual-process theory. Adv. Psychol. Sci. 31, 274–287. doi: 10.3724/SP.J.1042.2023.00274

[ref53] Yin H. C. Song X. Q. Yu Y. (2009). Development and validation of an athlete identity measurement tool. Sport Sci. 29, 61–64. doi: 10.16469/j.css.2009.08.007

[ref54] Zhang G. L. Cheng H. Li M. Z. (2019). The impact of after-hours electronic communication on employee proactive behavior. Manag. Rev. 31, 154–165. doi: 10.14120/j.cnki.cn11-5057/f.2019.03.014

[ref55] Zhang L. C. Gao S. Q. Zheng C. H. (2019). Research progress on physiological, psychological and neural mechanisms of ego depletion. Psychol Tech Appl. 7, 436–443. doi: 10.16842/j.cnki.issn2095-5588.2019.07.008

[ref56] Zhang Y. Tang N. (2017). Bayesian empirical likelihood estimation of quantile structural equation models. J. Syst. Sci. Complex. 30, 122–138. doi: 10.1007/s11424-017-6254-x, 41159878

[ref57] Zhang X. L. Zhang Y. X. Dong L. H. Du X. Y. (2022). Construction of a theoretical system of factors influencing social integration of Chinese retired athletes based on grounded theory. J. Xi'an Phys. Educ. Univ. 39, 574–583. doi: 10.16063/j.cnki.issn1001-747x.2022.06.009

[ref58] Zhiping G. Anmin L. Lin Y. (2017). Neural efficiency of athletes’ brain during visuo-spatial task: an fMRI study on table tennis players. Front. Behav. Neurosci. 11:72. doi: 10.3389/fnbeh.2017.00072 28491026 PMC5405064

[ref59] Zhu Y. Lyu Y. Wang Y. F. Wang L. X. (2018). How does coaching leadership influence employee innovation? Cross-level moderated mediation effects. Acta Psychol. Sin. 50, 327–336.

[ref60] Zhu S. L. Yuan T. J. Long L. R. (2022). How to enhance employee creativity in differentiated organizational change contexts: configuration analysis based on creativity interaction theory [in Chinese]. Sci. Technol. Progress Policy. 39, 141–150. doi: 10.6049/kjjbydc.2021050367

